# An informal logic of feedback-based temporal control

**DOI:** 10.3389/fnhum.2022.851991

**Published:** 2022-07-29

**Authors:** Sam Tilsen

**Affiliations:** Cornell Phonetics Lab, Department of Linguistics, Cornell University, Ithaca, NY, United States

**Keywords:** articulation, articulatory timing, speech rate, motor control, feedback, dynamical systems, phonology, prosody

## Abstract

A conceptual framework and mathematical model of the control of articulatory timing are presented, in which feedback systems play a fundamental role. The model applies both to relatively small timescales, such as within syllables, and to relatively large timescales, such as multi-phrase utterances. A crucial distinction is drawn between internal/predictive feedback and external/sensory feedback. It is argued that speakers modulate attention to feedback to speed up and slow down speech. A number of theoretical implications of the framework are discussed, including consequences for the understanding of syllable structure and prosodic phrase organization.

## Introduction

Perhaps you have been in a situation in which it was necessary to *shush* someone. For example, imagine you are reading in a library, when a rude person nearby begins talking on their cell phone. You glare at them and say “shhh,” transcribed phonetically as [∫::]. What determines the duration of this sound? Consider now a different situation: in a coffee shop you are ranting to your friend about the library incident, and your friend tells you to slow down because you are talking too fast. You take a deep breath and proceed more slowly. How do you implement this slowing? The focus of this manuscript is on how variation in the temporal properties of event durations (your “shhh”) and variation in event rate (your rapid coffee shop rant) are related. More specifically, what is the mechanistic connection between control of event timing on short timescales and control of speech rate on longer timescales? It is argued that the answer to this question involves a notion of feedback, and that the same feedback mechanisms are involved on both timescales. In other words, control of event timing involves feedback, and control of rate is reducible to control of timing.

**FIGURE 1:**
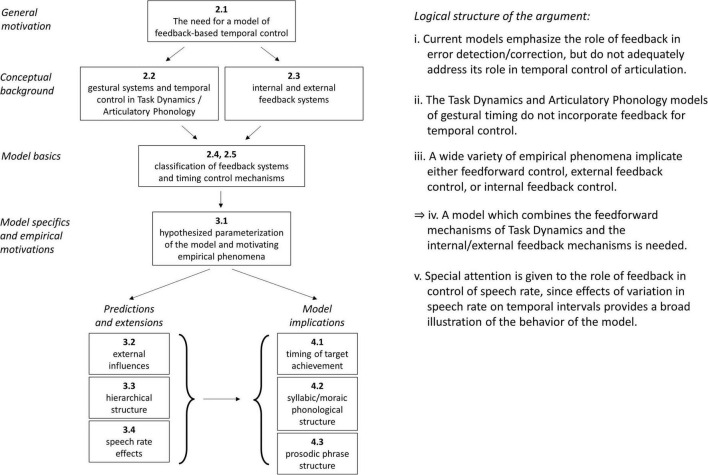
Graphical illustration of manuscript organization and propositions comprising the main argument.

Figure 1 provides a graphical depiction of the organization of the manuscript, and lays out the logical structure of the main argument. The motivation for developing a feedback-based model of temporal control is based on three propositions: (i) That current models generally do not provide an empirically adequate account of the role of feedback in the temporal control of articulation (see Section “The need for a model of feedback-based temporal control”). (ii) That the Task Dynamics (TD)/Articulatory Phonology (AP) framework does not use feedback for temporal control (see Section “Gestural systems and temporal control of gestural activation”). (iii) That empirical phenomena require internal/external feedback control, as well as feedforward control (see Section “Model space and hypotheses”). From these propositions it follows (iv): a model that combines the gestural mechanisms of TD with internal and external feedback-based temporal control is needed. It is important to emphasize that temporal control—control of the timing and relative timing of events—is different from the issue of how movement events are controlled once an intention to achieve articulatory/auditory goals is assumed to be present. The section “The need for a model of feedback-based temporal control” argues that existing models of feedback focus on how tasks/goals are translated to movements but not on how tasks/goals are organized in time. The section “Gestural systems and temporal control of gestural activation” introduces the gestural framework of TD along with the central topic of this manuscript: the question of what causes articulatory events to begin and to end? The section “External feedback vs. internal feedback” defines the notions of internal and external feedback which are employed throughout this manuscript and the sections “Time-representing systems and timing control” and “Deterministic behavior of TiRs and effects of stochastic forces” classify and demonstrate the basic properties of the systems which are used for temporal control in the proposed model. The specific hypotheses of the model and the empirical phenomena which motivate them are detailed in the section “Model space and hypotheses.” Further predictions and extensions of the model are discussed in the sections “External influences on parameters,” “Parallel domains of competitive selection,” and “A model of speech rate control with selectional effects.” Finally, some important implications of the model are discussed in the section “General discussion,” regarding the control of timing of target achievement (see Section “No direct control of the timing of target achievement”), syllabic and moraic phonological structure (see Section “Reinterpretation of syllabic and moraic structure”), and prosodic phrase structure (see Section “Reinterpretation of prosodic phrase structure and boundaries”).

**FIGURE 2:**
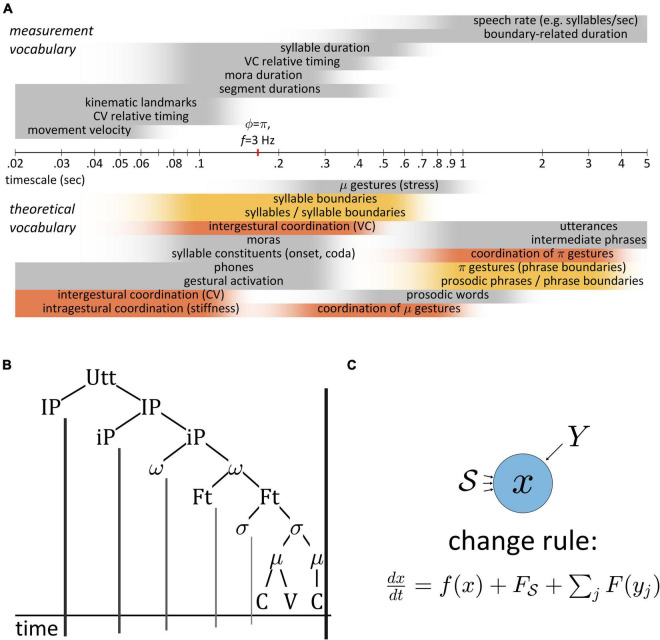
**(A)** Comparison of timescales associated with various measurements and theoretical constructs used to conceptualize temporal patterns. Time axis is logarithmic. Shaded intervals approximately represent ranges of time in which terminology applied. **(B)** Hierarchical conception of prosodic structure and implicit projection of units to boundaries in a temporal coordinate. **(C)** Generic system schema, where change in the state variable *x* is a function of *x* itself and of forces from the surroundings *S* and from other systems *Y*.

Temporal patterns in speech are challenging to characterize because they exist across a wide range of analysis scales. Figure 2A shows rough approximations of timescales associated with various measurements and theoretical vocabularies. Even over the modest range of 20 ms to 5,000 ms (shown in a logarithmic axis), there are many different ways to associate time intervals with theoretical constructs. Furthermore, there are certain terms—“coordination,” “boundaries”—which reappear across scales, and problematically necessitate different interpretations.

It is rarely the case that models of small scale phenomena, such as articulatory timing within syllables, are integrated with models of larger scale phenomena, such as boundary-related slowing. One noteworthy exception is the *π-gesture* model (Byrd and Saltzman, 2003), which modulates the rate of a global dynamical clock in the vicinity of phrase boundaries, thereby slowing the timecourse of gestural activation. Another example is the multiscale model of Tilsen (2013), where oscillator-based control of gestural timing is limited to syllable-sized sets of gestures that are competitively selected with a feedback-based mechanism. This early combination of oscillator- and feedback-based control led to the development of Selection-Coordination theory (Tilsen, 2014, 2016), an extension of the AP framework that uses feedback control to account for a variety of cross-linguistic and developmental patterns. A recent proposal in this context is that speech rate is controlled by adjusting the relative contributions of external (sensory) feedback and internal (predictive) feedback (Tilsen, 2018). One of the aims of this manuscript is to elaborate on this idea, advancing that > the generalization that temporal control in speech is largely (but not exclusively) feedback-based. This aim is also the primary novelty of the manuscript: it argues directly that internal and external feedback systems, beyond their commonly held roles in state estimation and error detection/correction, play a fundamental role in the control of timing. In a more general sense, the novelty of the manuscript is its original combination of feedforward control mechanisms described in AP and TD (Saltzman and Munhall, 1989; Saltzman et al., 2008) with feedback control mechanisms used in competitive queuing models (Grossberg, 1987; Bullock and Rhodes, 2002), while explicitly distinguishing internal and external feedback.

A broader aim is to argue for a worldview in which speech patterns are understood to result from interactions of dynamical systems. The “informal logic” developed here advocates for new way of thinking about patterns in speech. It is relevant both for the study of speech motor control, specifically in relation to feedback and control of timing, and for theories of phonological representation, sound patterns, and change. The informal logic challenges the prevailing ontologies of many phonological theories by rejecting the notion that speech is cognitively represented as a structure of hierarchically connected objects, as in Figure 2B. It also rejects the notion that such units project “boundaries” onto the temporal dimension of the acoustic signal. Most importantly, the logic holds that speakers never control event durations directly: rather, durational control is accomplished *via* a class of systems which *indirectly* represent time. They do this by integrating the forces they experience from other systems, or from their surroundings.

The systems-oriented approach can provide a more coherent understanding of temporal phenomena across scales. Its logic is qualified as “informal” because, unlike a formal logic, it does not rely heavily on symbolic forms; rather, the schemas presented below are iconic and indexical, designed to help users rapidly interpret complex patterns of system interactions. At the same time, the schemas can be readily mapped to a explicit mathematical model. All model equations and simulation details are described in Supplementary Material, and all code used to conduct simulations and generate figures has been made available in a public repository, here: https://github.com/tilsen/TiR-model.git. The model has been designed to be as simple as possible while being able to generate a variety of temporal patterns. All model equations are presented in the Supplementary Material rather than the manuscript, for three reasons. First, the manuscript itself is geared toward a larger audience of readers who are interested in a conceptual understanding of the framework and its applications; hence a graphical rather than symbolic approach to illustrating model structure has been adopted throughout. Second, for the subset of readers who are interested in understanding the mathematical implementation, presenting the equations together in the Supplementary Material facilitates this endeavor. Third, in the case of modeling complex cognitive systems, it is important in general not to overemphasize the specific details of mathematical models; following Saltzman and Munhall (1989), I believe that “the primary importance of the work lies not so much in the details of [the] model, but in the problems that can be delineated within its framework” (Saltzman and Munhall, 1989, p. 335). My hope is therefore that the informal logic presented here can be used by researchers to conceptualize empirical phenomena, without requiring them to implement the model I have constructed. And yet, for those who are interested in critiquing or improving the model, or adapting it to interface with other models, I have made substantial efforts to facilitate this. Finally, although the implications of the framework are fairly general, it is nonetheless narrowly focused on describing a logic of *temporal* control. Issues related to “spatial” dimensions of feedback or to feedback modalities are set aside for future extensions.

## Background

Below we argue that existing models of speech production do not adequately account for the temporal control of articulatory events, and hence there is a need for a model that focusses on temporal patterns in speech. Subsequent sections introduce some of the key concepts that are incorporated in the model developed here. As a general principle, the components of the model are always viewed as systems and their relations are viewed as interaction forces. Systems are abstract entities which have time-varying internal states. Our analytical task is to formulate change rules to describe how the system states evolve over the course of an utterance, as shown generically in Figure 2C. This setup provides a frame in which to analyze and interpret the causes of empirical patterns in speech. Moreover, to draw generalizations about systems and their interactions we must classify them. To accomplish this in the following sections we define terms below such as *internal*, *external*, *feedback*, and *sensory*. These terms are necessarily relative and therefore potentially ambiguous out of context, thus the reader should pay careful attention to these definitions to avoid confusion.

### The need for a model of feedback-based temporal control

The motivation for the model developed here is that there currently exists no model of speech motor control that provides an empirically adequate account of articulatory event timing. Importantly, the issue of event timing is different from the issue of how movement is controlled when an intention to generate movement is presupposed. There are several models that provide accounts of how movements are controlled, but only when it is assumed that a speaker has an intention to achieve some goal—these are models which focus on control *from* an intention. As discussed below, most of these models do not address how the intentions themselves are organized in time, i.e., the control *of* (the timing of) intentions. Only one of the models provides explicit mechanisms for governing the temporal organization of intentions, but that model is inadequate from an empirical perspective. By “intention” here I mean the aim of a speaker to achieve a goal-related outcome(s). This abstract term is used in order to generalize over models that are based on different hypothetical entities—often either phones/phonemes or tasks/articulatory gestures.

It is crucial for the reader to understand that control *from* intentions and control *of* intentions are distinct topics: most speech motor control models assume that intentions to conduct movement exist, and ask how those movements are realized and modulated by feedback; in contrast, my interest in this manuscript is what causes the intentions themselves to begin and to end. For example, the questions asked by researchers interested in the control *of* intentions (e.g., Guenther and Hickok, 2016) are “What exactly are the goals, or targets, of the speech production planning process?” and how can the nervous system “generate a complex muscle activation pattern that satisfies the goals of the movement”? These are important questions but they are not the focus of this manuscript, because they are not about the temporal organization of the goals/targets of speech.

Indeed, most speech motor control models do not adequately address the question of temporal control. First, consider the directions into velocities of articulators (DIVA) model (Tourville and Guenther, 2011). In the relatively recent description of this model in Guenther and Hickok (2016), it is stated that “the DIVA model’s feedforward commands constitute a form of motor program and, as such, they are closely related to the concepts of a gestural score”; the authors then state that “a gestural score is a stored representation of properly timed vocal tract gestures.” It is held that—following (Levelt and Wheeldon, 1994)—frequently used syllables or sequences of syllables are stored as motor programs, and infrequent syllables may be assembled during speech planning from phoneme-sized programs. This characterization of a gestural score as “a stored representation of properly timed vocal tract gestures” is inconsistent both with early formulations of the TD model of speech production, as well as most of the recent theoretical literature on AP and TD (Browman and Goldstein, 1989; Saltzman and Munhall, 1989), which holds that patterns of gestural activation are generated online rather than being stored. This point is discussed more thoroughly in Section “The need for a model of feedback-based temporal control,” in the context of a close examination of TD model. Ultimately, the DIVA model alone does not specify what determines the timing of its feedforward commands; rather it presupposes that some timing pattern is already specified.

The gradient ordering (GODIVA) model (Bohland et al., 2010) is an extension of DIVA that incorporates a model of timing, yet this model is empirically inadequate in several ways. GODIVA employs a competitive queuing mechanism to sequentially activate the individual phonemes that are hypothesized to comprise a syllable. Once a syllable is selected, the plan for the first phoneme of that syllable becomes active for a “short duration” (parameter τ of Equation 6 in Bohland et al., 2010), and each subsequent phoneme instantaneously becomes active upon the deactivation of the preceding one. Hence, the model provides a purely sequential account of the temporal organization of intentions (i.e., the goals associated with phonemes). GODIVA is empirically inadequate for several reasons, which are briefly mentioned here and discussed more thoroughly in Section “Model space and hypotheses.” First, articulatory movements in adult speech overlap substantially, especially in syllable onsets, where movements associated with consonantal constrictions are largely coextensive in time with vowel-related movements. The GODIVA model does not explain how such extensive temporal overlap could arise from plans which are selected sequentially; in actuality, it predicts the opposite: that consonantal and vocalic movements should occur in a non-overlapping sequence. Second, in complex-onset syllables such as CCV, the order in which the constriction formation movements are initiated empirically is such that the initiation of vocalic movement intervenes between the initiations of the constriction formations: thus GODIVA explicitly imposes a CCVCC sequencing of phones within syllables that does not correspond to the order in which movements are initiated in empirical data (see Section “Empirical motivation for pre-vocalic oscillator-based control” for references). Third, the model does not does not discuss sources of variation in the phoneme duration parameter τ, and therefore it is hard to say what it predicts regarding variability in event durations. Finally, the model does not provide a role for sensory feedback to influence the timing of phone selection; instead, the role of sensory feedback in DIVA/GODIVA is limited to the detection and correction of errors, which can only indirectly influence timing.

The hierarchical state feedback control (HSFC) model of Hickok (2012) argues that both external and internal sensory feedback are used for the detection and correction of errors in speech plans and their outputs. However, the model focuses on the activation of hypothesized syllable and phoneme motor programs; it does not generate articulatory events. Indeed, the words “duration” and “timing” are never used to describe model-generated events in Hickok (2012). As with DIVA/GODIVA, HSFC focuses on the use of feedback for error detection/correction, but not on the temporal organization of the intentions to achieve targets. The equilibrium point model of motor control (Feldman, 1986; Feldman and Levin, 2009) is also not a model of temporal control; it describes how goals (changes in equilibria) are implemented through effector/muscle synergies. This model does not address the issue of when changes in equilibrium points occur. Similarly, the powerful feedback-aware control of tasks in speech (FACTS) model (Parrell et al., 2018, 2019), although avoiding the empirical problems associated with phoneme-sequence conceptions of speech, is a model of how control is achieved given a presupposed temporal pattern of intentions. FACTS does not aim to address how the temporal pattern of intentions is generated in the first place.

Thus, many speech motor control models—DIVA, HSFC, FACTS, equilibrium points—do not directly address the role of feedback in temporal control; instead, they employ feedback for error detection/correction. The GODIVA model, which contains a mechanism for the sequencing of phones, does not allow feedback any direct role in this sequencing process, and imposes an ordering of phones that is not empirically motivated.

### Gestural systems and temporal control of gestural activation

**FIGURE 3:**
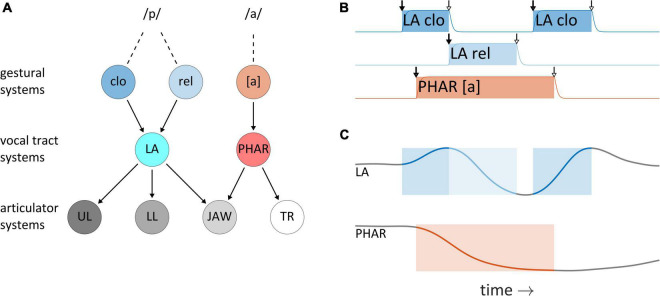
System organization and interactions in the Task Dynamics model. **(A)** Organization of system interactions (see text for descriptions of systems). **(B)** Gestural activation intervals for the CVC syllable pop. **(C)** Vocal tract geometry changes resulting from the actions of gestural systems on vocal tract systems. Lip aperture (LA) and pharyngeal constriction (PHAR) timeseries are shown.

This section describes the understanding of articulatory control adopted here, which originates from TD (Kelso et al., 1986; Saltzman and Munhall, 1989). It is argued that although TD provides a useful framework for thinking about temporal control, the model and its phonological counterpart AP (Browman and Goldstein, 1989) leave important questions regarding articulatory timing unresolved; most importantly, they do not make use of feedback for control of timing. In TD, changes in the physical outputs of speech—vocal tract shape and distributions of acoustic energy—are indirectly caused by systems called *articulatory gestures*. Figure 3A schematizes the organization of system interactions in the TD model for production of the word *pop*: gestural systems for bilabial closure (clo), bilabial release (rel), and the vocalic posture of [a] exert driving forces on vocal tract systems of lip aperture (LA) and pharyngeal constriction degree (PHAR), which in turn exert forces on articulator systems for the upper lip (UL), lower lip (LL), jaw, and tongue root (TR). [As an aside, note that the framework attributes no ontological status to phones or phonemes—these are merely “practical tools” (Browman and Goldstein, 1990) or inventions of scientific cultures (Ladefoged, 2001; Port and Leary, 2005).] Gestural system states are defined in normalized activation coordinates which range from zero to one, and gestures are understood to abruptly become active and subsequently deactivate, as shown for the word *pop* in Figure 3B—this panel includes the activation intervals of a bilabial closure gesture (LA clo), a bilabial release gesture (LA rel), and a tongue root gesture, which achieves a pharyngeal constriction for the vowel [a]. When their activation is non-zero, gestures exert forces on vocal tract systems, which can lead to movement, as shown in Figure 3C for timeseries of lip aperture (LA) and pharyngeal constriction degree (PHAR).

In both a theoretical and technical sense, gestures should be understood as *systems*. They are entities which have internal states and which experience and exert forces. Accordingly, gestures are not movements, nor are they periods of time in which movements occur. To reinforce this point we often refer to them (redundantly) as *gestural systems*. The distinction is important because it is common to refer to movements of vocal organs as “gestures”—but this can cause confusion. Similarly, the periods in which gestural systems obtain states of high activation (shaded intervals in Figure 3B) are sometimes called “gestures”—these periods are better described as *gestural activation intervals*. The point here is simply that metonymic extensions of “gesture” to refer to physical movements or activation intervals should not be conflated with the systems themselves. Furthermore, the vocal tract and articulator system states of the TD model are nervous system-internal representations of the physical geometry of the vocal tract/effectors. The actual geometry of the vocal tract is not modeled explicitly in TD and can in principle diverge from these internal representations. Finally, in the TD model, vocal tract system states are defined in position, velocity coordinates, and interactions between gestural systems and vocal tract systems are analogous to mechanical forces. These particular analogies do not apply to forces experienced by gestural systems, nor to other types of systems which we develop below. The systems we construct are better analogized to many-body, open thermodynamic systems: their “activation” states are conceptualized as energies, rather than positions/velocities, and their interactions are analogized to thermodynamic generalized forces. This set of conceptual metaphors is further discussed in the Supplementary Material, in the context of the model equations.

The TD framework is particularly valuable because it clarifies the questions that must be addressed in order to understand temporal patterns in speech. There are two questions of paramount importance regarding temporal control: (i) What causes inactive gestural systems to become active? and (ii) What causes active gestural systems to become inactive? These questions correspond to the arrows marking initiations and terminations of the gestural activation in Figure 3B.

(i) *What causes gestures to become active?* In answering this question, we temporarily adopt the perspective that the entire set of gestures is a “system.” One possible answer then is that there are some *external* systems which exert forces on the gestures. By “external” we mean systems which are “outside” of the set of gestures, and we refer to such systems as *extra-gestural*. Another possibility is that the gestural systems experience forces from each other, in which case the activating forces come from “inside of the system” or are *internal* to the system of gestures, i.e., *inter-gestural*. Note that the first gesture to become active must necessarily be activated by an extra-gestural system, because there is presumably no way for a gestural system to spontaneously “activate itself” or to be activated by inactive gestural systems.
(ii) *What causes gestures to cease to be active?* The extra-gestural and inter-gestural forces described above are both plausible sources of deactivation. A third possibility, unavailable in the case of activating forces, is that deactivation is caused by actions of individual gestural systems on themselves, i.e., *intra-gesturally*. We elaborate below on how this differs from inter-gestural control.

The TD model of speech production developed by Saltzman and Munhall (1989) did not resolve which of the various sources of initiating and terminating forces are utilized. Saltzman and Munhall heuristically hand-specified activation intervals to fit empirical data, but they proposed that the model could be extended with the serial network of Jordan (1986) to dynamically control gestural activation. In this serial network, the hidden layers responsible for sequencing might be interpreted as extra-gestural forces. Much attention has been given to the issue of gestural timing in the framework of AP (Browman and Goldstein, 1986, 1988, 1990, 1992). Many early descriptions of timing in AP—in particular references to “phasing”— imply that initiating forces are inter-gestural and that terminating forces are intra-gestural, in line with the explicit interpretations of phasing in Kelso and Tuller (1987). In contrast, later descriptions hypothesize that gestures are activated by a separate system of gestural planning oscillators (Goldstein et al., 2006; Saltzman et al., 2008), which are extra-gestural. These approaches attribute no role to feedback in the initiation or termination of gestures. Thus the current situation is one in which several different possible understandings of feedforward temporal control of gestures have been proposed, none of which specifically implicate feedback.

To summarize, the systems-view of gestural control in the TD framework provides two generic options for what causes gestures to become active or cease to be active—extra-gestural systems or other gestures (inter-gestural control)—along with a third option of intra-gestural control as a form of self-deactivation. There is no theoretical consensus on which of these are actually involved in control of articulatory timing, or in what contexts they may be utilized. Furthermore, feedback has not been incorporated into this framework for the purpose of controlling gestural timing.

### External feedback vs. internal feedback

Definitions of external and internal feedback are presented here. The term *feedback* has a variety of different uses. Here *feedback* refers to information which—in either a direct or indirect manner—is produced by some particular system, exists outside of that system, and subsequently plays a role in influencing the state of that same system. Thus feedback is always defined relative to some reference system. In current contexts the reference system is sometimes a particular gestural system, other times the entire set of gestural systems, and most often the central nervous system. Feedback in this sense is a very general notion, and does not presuppose that “sensory” organs such as the cochlea or muscle stretch receptors are involved.

Note also that the “information” referred to in the above definition of feedback can be plausibly given a technical interpretation (Shannon, 1948), but the actual quantification of said information faces many obstacles. Strictly speaking, information is produced when an observer’s uncertainty in the state of a system is reduced. Quantification of information production requires knowledge of the probability distribution over states of an observed system, along with definition of the observed and observing systems. For example, a vocal tract system “observes” the forces it experiences from a gestural system, but to quantify the information produced by this observation we need a probability distribution over all possible gestural system forces. However, to simply determine whether information meets the definition of feedback, we need only to identify the chain of interactions associated with information production. If that chain forms a loop back to the particular system of interest, then it meets the definition of feedback.

**FIGURE 4:**
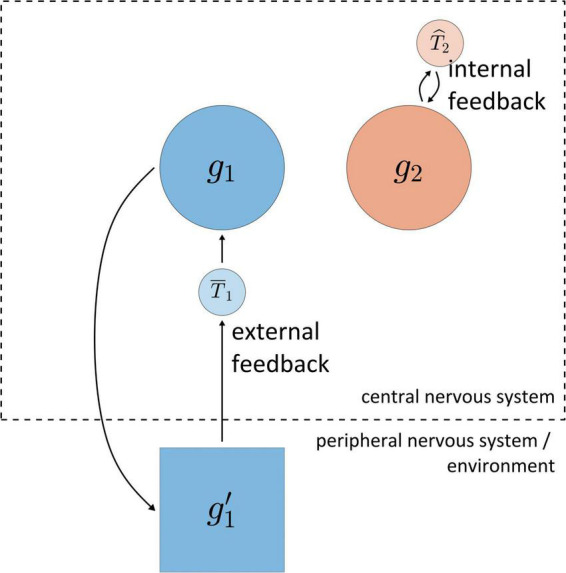
Schematic illustration of distinction between internal and external feedback. The dashed line represents the boundary of the central nervous system. Systems *g*_1_ and *g*_2_ are gestural systems, *g*’_1_ is system which represents information associated with *g*_1_ outside of the central nervous system, and *T*_1_ and *T*_2_ are hypothetical systems which use feedback to act on *g*_1_/*g*_2_.

For a logic of feedback-based temporal control of speech it is crucial to distinguish between *external feedback* and *internal feedback*, as illustrated in Figure 4. The reference system is the central nervous system (CNS, consisting of cortex, brainstem, and spinal cord). External feedback involves information that (i) is originally generated within the CNS, (ii) causes information to be produced outside of the CNS, and (iii) in turn causes information to be produced within the CNS; correlations must obtain between the information in these three stages. For example, activation of the gestural system *g*_1_ causes the production of various forms of information in the environment (movement of articulators, generation of acoustic energy), which is in turn transduced in the peripheral nervous system (depolarization of hair cells in the cochlea and sensory muscle fibers) and subsequently produces information in cortical systems. For current purposes we draw no distinctions between various sensory modalities, which are lumped together as system *g*′_1_ in Figure 4. The information associated with *g*′_1_ can ultimately influence the state of *g*_1_, and hence meets our definition of feedback. Notice that Figure 4 includes a system labeled ${\bar{T}}_{1}$, which uses the external feedback from *g’*_1_ to act on *g*_1_.

In contrast to external feedback, internal feedback is information which never exists outside of the CNS. For example, in Figure 4 the gestural system *g*_2_ generates information that system ${\hat{T}}_{2}$ uses to act on *g*_2_. Thus the contrast between external and internal feedback is based on whether the relevant information at some point in time exists “outside of”/“external to” the central nervous system. External feedback may be also described as “sensory” feedback, but with a caveat: one could very well also describe internal feedback as “sensory,” in that any experience of force—regardless of its origins—can reasonably be considered a form of *sensation*. The point is simply that the word “sensory” is ambiguous regarding what is being sensed, and so the qualifiers *internal* and *external* are preferred, with the CNS being the implied reference system. Internal feedback can also be described as “predictive,” but we should be cautious because this term strongly evokes an agentive interpretation of systems.

The distinction between external and internal feedback is only partly orthogonal to the distinction between extra-gestural, inter-gestural, and intra-gestural control. The full system of gestures is by definition within the CNS; hence feedback associated with inter-gestural and intra-gestural control is by definition internal feedback. In contrast, extra-gestural control may involve either external feedback (e.g., auditory or proprioceptive information) or internal feedback from CNS-internal systems. This can be confusing because “extra”-gestural control does not entail external feedback—hence the necessity to keep tabs on the system boundaries to which our vocabulary implicitly refers. When describing feedback, the reference system is the CNS. When describing control of gestural activation, the reference system is either the full system of gestures (for extra-gestural control) or individual gestural systems (for inter- vs. intra-gestural control).

The Task Dynamic model incorporates no feedback of any form for gestural systems. Nonetheless, Saltzman and Munhall cited the necessity of eventually incorporating sensory feedback, stating: “without feedback connections that directly or indirectly link the articulators to the intergestural level, a mechanical perturbation to a limb or speech articulator could not alter the timing structure of a given movement sequence” (Grossberg, 1987, p. 360). Note that here Saltzman and Munhall expressed a concern with the *temporal* effects of perturbation rather than *spatial* effects—in this manuscript, we are similarly focused on timing but recognize that a complete picture should incorporate a fully embodied and sensorially differentiated model of the articulatory and acoustic dimensions of feedback.

### Time-representing systems and timing control

To augment our classification of the ways in which gestural systems may be activated or deactivated, we need to think about how time may be “measured,” “estimated,” or “represented” by the nervous system. Researchers have adopted various ways of talking about different types of systems that serve this function (Kelso and Tuller, 1987; Schöner, 2002)—timers, clocks, timekeepers, virtual cycles, etc., with the discussion of Schöner (2002) being particularly informative. For current purposes, we describe such systems as “time-representers” (TiRs) and develop a multidimensional classification. Despite this name, we emphasize that temporal representations are *always indirect*: the states of TiR systems are never defined in units of time.

**FIGURE 5:**
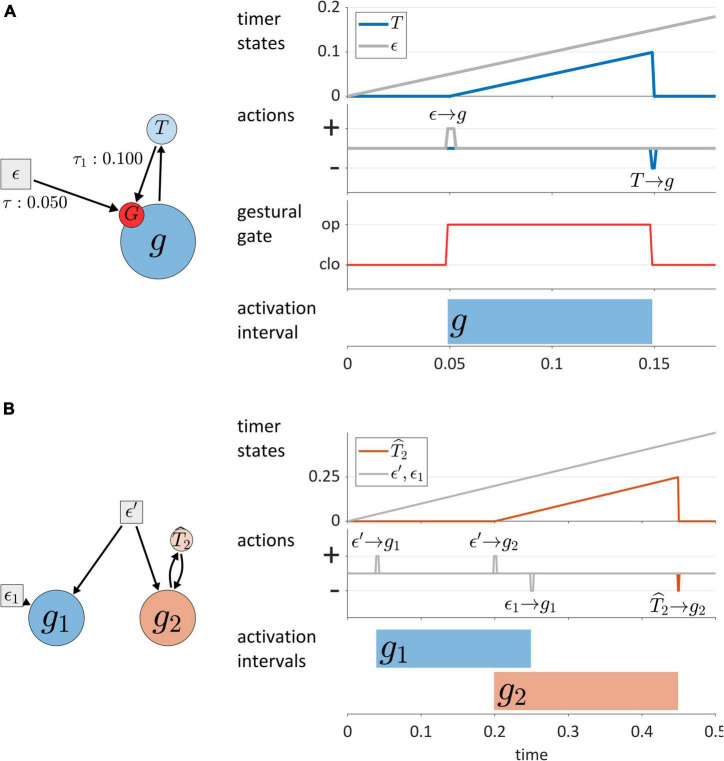
**(A)** Model of interactions between gestures and TiRs, with depiction of the gestural gating system G that TiRs act upon. Panels on the right show timer states, timer actions on gestures, gestural gating system states, and gestural activation interval. **(B)** Distinction between autonomous TiRs (ϵ′, ϵ_1_) and non-autonomous TiRs (${\hat{\text{T}}}_{2}$).

Before classifying TiRs, we make a couple points regarding their interactions with gestures. First, each gestural system is associated with a gating system, labeled “G” in Figure 5A. The gating system states are treated as binary: gates are either open or closed. When a gestural gate is open, the activation state of the associated gestural system transitions rapidly toward its normalized maximum activation of 1. Conversely, when the gate is closed, the gestural system transitions rapidly toward its minimum value. For current purposes, transitions in gestural activation states occur in a single time step, as in Saltzman and Munhall (1989). Nothing hinges on this simplified implementation and the model can be readily extended to allow for activation ramping or non-linearities to better fit empirical tract variable velocity profiles (Sorensen and Gafos, 2016).

Second, TiRs act on gestural gating systems, not directly on gestures, and thus function to activate/deactivate gestural systems indirectly. One reason for including gating systems as intermediaries between TiRs and gestures is that they allow for the dynamics of gestural systems to be dissociated from the forces that control gestural activation. The actions of TiRs are modeled as brief, pulse-like forces, and always depend on TiR-internal states: each TiR has threshold parameters (τ) which specify the internal states (in units of activation) at which the TiR acts on gating systems. The action threshold parameters are labeled on the arrows of Figure 5A. To reduce visual clutter in model schemas, gating systems are omitted from subsequent figures.

One main dimension of TiR classification involves whether a TiR is autonomous or non-autonomous. An *autonomous* TiR does not depend on either gestural or sensory system input to maintain an indirect representation of time. Figure 5B shows two examples of autonomous TiRs. The first is ϵ′, which activates gestures *g*_1_ and *g*_2_. The second is ***ϵ*_***1***_**, which deactivates *g*_1_. Note that autonomous TiRs *do* require an external input to begin representing time—they need to be “turned on”/de-gated—but subsequently their state evolution is determined by a growth rate parameter. This parameter may vary in response to changes in a hypothesized “surroundings” or contextual factors.

In contrast to autonomous TiRs, the states of *non-autonomous* TiRs depend on input from a gestural or sensory system. Non-autonomous TiRs integrate the forces that they experience from a given system. An example is ${\hat{\text{T}}}_{2}$ in Figure 5B, which receives input from *g*_2_ and deactivates *g*_2_ upon reaching a threshold state of activation, here τ = 0.25. Non-autonomous TiRs are associated with integration rate parameters α, which determine how much the forces they experience contribute to changes in their internal states.

The key difference between autonomous TiRs and non-autonomous ones is that the states of the autonomous TiRs evolve independently from the states of gestures or sensory systems. In the example of Figure 5B the states of autonomous TiRs ϵ′ and ***ϵ*_***1***_** are assumed to be 0 at the beginning of the simulation and increase linearly in a way that represents elapsed time. In this example (but not in general), the growth rates of autonomous TiR states were set to 1/Δ*t* (where Δ*t* is the simulation time step); consequently, their activation states exactly correspond to elapsed time. This is convenient for specifying threshold parameters that determine when TiRs act on other systems. Similarly, the integration rate parameters of non-autonomous TiRs were parameterized to represent the time elapsed from the onset of gestural activation. In general, the correspondence between TiR activation values and elapsed time is neither required nor desirable, and we will see how changes in TiR growth rates/integration rates are useful for modeling various empirical phenomena.

**FIGURE 6:**
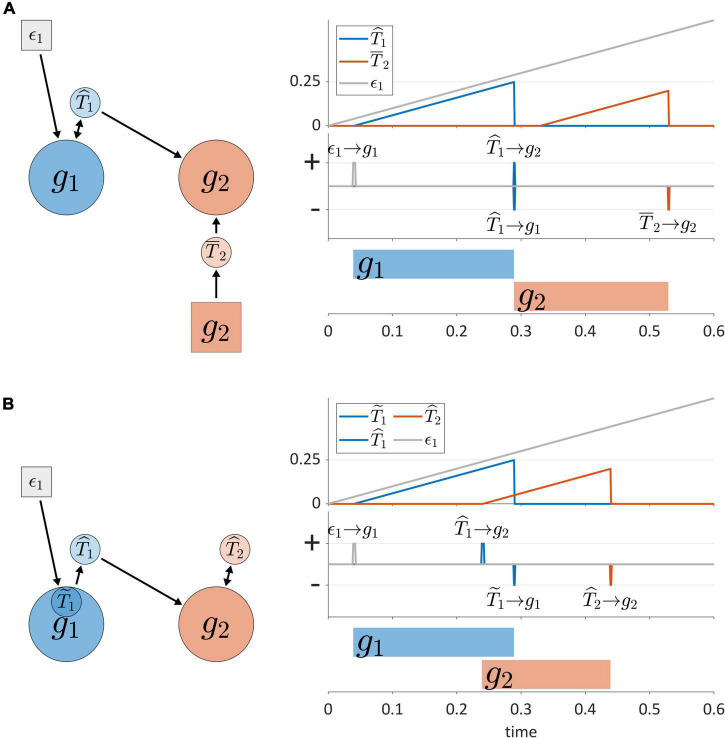
**(A)** External vs. internal sources of feedback for non-autonomous TiRs. Panels on the right show timer states, timer actions, and gestural activation intervals. **(B)** Example of inter-gestural vs. isolated/intra-gestural TiRs.

Another dimension of TiR classification involves the sources of input that non-autonomous TiRs make use of to represent time. Non-autonomous TiRs can be described as *external* or *internal*, according to whether they integrate external or internal feedback. This distinction is illustrated in Figure 6A, where the non-autonomous TiR ${\hat{\text{T}}}_{1}$ can be described as internal because it integrates feedback directly from gesture *g*_1_. In contrast, the non-autonomous TiR ${\bar{\text{T}}}_{2}$ is external because it integrates feedback from sensory systems which encode the actions of *g*_2_ outside of the CNS.

Non-autonomous, internal TiRs are further distinguished according to whether they are inter-gestural or intra-gestural (internal to a gesture). Intra-gestural internal TiRs can only act on the particular gestural system that they are associated with, and can integrate forces only from that gesture. Inter-gestural TiRs can act on and experience forces from any gestural system. For example, in Figure 6B, the deactivation of *g*_1_ is controlled by an intra-gestural TiR ${\tilde{\text{T}}}_{1}$, but the inter-gestural TiRs ${\hat{\text{T}}}_{1}$ and ${\hat{\text{T}}}_{2}$ activate and deactivate *g*_2_, respectively. The distinction is useful if we wish to impose the condition that a TiR is isolated from all systems other than a particular gesture.

The distinction between inter-gestural and intra-gestural TiRs can be viewed in relation to different aspects of the virtual cycles that Kelso and Tuller (1987) proposed to govern gestural timing. Tuller and Kelso held that each gesture could be associated with a virtual cycle, which might be described as a “single-shot” oscillation. Different phases of the cycle were hypothesized to correspond to events such as gesture initiation, achievement of maximum velocity, target achievement, and gesture termination. It was suggested in Browman and Goldstein (1995) that when a virtual cycle phase of 3π/2 rad (270°) is reached, a gesture is deactivated. In this regard intra-gestural TiRs can implement the functions of virtual cycles: their activation states can be converted to a normalized coordinate that ranges from 0 to 2π, and their growth rates can be adjusted to match the natural frequency of an undamped harmonic oscillator. However, Kelso and Tuller (1987) also proposed that intergestural timing might involve specification of the initiation of the virtual cycle of one gesture relative to the virtual cycle of another. Only inter-gestural TiRs can serve this function, because unlike intra-gestural TiRs, they can act on gestural systems that they are not directly associated with. For all of the purposes that follow in this manuscript, intra-gestural TiRs are unnecessary and we make use of inter-gestural TiRs instead.

**FIGURE 7:**
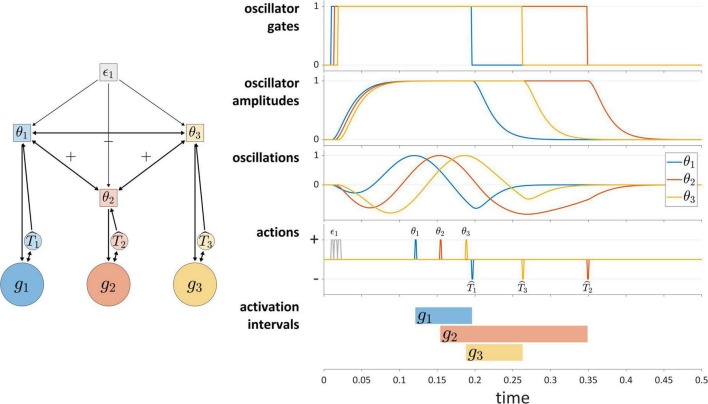
The coupled oscillators model in the TiR framework. Periodic TiRs θ1, θ2, and θ3 are phase coupled as indicated by +/– symbols. The oscillator gates, radial amplitudes, and oscillations (amplitude × cosine of phase) are shown. Due to the pattern of phase coupling imposed here, initiation of gestural systems *g*_1_ and *g*_3_ are symmetrically displaced from initiation of *g*_2_.

Autonomous TiRs can differ in whether their state evolution is aperiodic or periodic. Periodic (or technically, quasi-periodic) TiRs are used in the coupled oscillators model (Saltzman et al., 2008), where each gesture is associated with an oscillatory system called a *gestural planning oscillator*. The planning oscillators are autonomous TiRs because they do not integrate gestural or sensory system states, as can be seen in Figure 7. They are often assumed to have identical frequencies and to be strongly phase-coupled, such that the instantaneous frequencies of the oscillators are accelerated or decelerated as a function of their phase differences. When a given planning oscillator reaches a particular phase, it “triggers” the activation of the corresponding gestural system. The “triggering” in our framework means that the TiR acts upon a gestural system, in the same way that other TiRs act upon gestural systems. The schema in Figure 7 illustrates a system of three periodic TiRs in which θ_1_ and θ_3_ are repulsively phase coupled to one another while being attractively phase coupled to θ_2_.

The phase coupling configuration in Figure 7 generates a pattern of relative phase that—*via* phase-dependent actions on gestural systems—leads to a symmetric displacement of initiations of gestures *g*_1_ and *g*_3_ relative to initiation of *g*_2_. Statistical tendencies toward symmetric displacement patterns of this sort are commonly observed in two phonological environments: in simple CV syllables, the initiations of constriction formation and release are displaced in opposite directions in time from the initiation of the vocalic gesture (Tilsen, 2017); in complex onset CCV syllables, the initiations of the first and second constriction are equally displaced in opposite directions from initiation of the vocalic gesture (Browman and Goldstein, 1988; Marin and Pouplier, 2010; Tilsen et al., 2012).

The coupled oscillators model has not been used to govern gestural deactivation. Furthermore, a gating mechanism is needed to prevent oscillators from re-triggering gestural systems in subsequent cycles or to prevent them from triggering gestures prematurely. To address this, in the current implementation each oscillator is described by three state variables: a phase angle, a radial amplitude, and the derivative of the radial amplitude. Furthermore, each oscillator is associated with a gating system that controls oscillator amplitude dynamics. As shown in Figure 7, intergestural TiRs close these oscillator gates. Moreover, a condition is imposed such that oscillators can only trigger gestural activation when their amplitudes are above a threshold value. The “oscillations” panel of Figure 7 shows a representation of oscillator states that combines phase and amplitude dimensions (the product of the amplitude and the cosine of phase). Further details are provided in the Supplementary Material.

An important hypothesis is that oscillator frequencies are constrained in a way that aperiodic TiR growth rates are not. We refer to this as the *frequency constraint hypothesis*. The rationale is that the oscillator states are believed to represent periodicity in a short-time integration of neuronal population spike-rates; this periodicity is likely to be band-limited due to intrinsic time-constants of the relevant neural circuits and neurophysiology. A reasonable candidate band is theta, which ranges from about 3–8 Hz (Buzsáki and Draguhn, 2004; Buzsaki, 2006), or periods of about 330 to 125 ms. On the basis of these limits, certain empirical predictions regarding temporal patterns can be derived, which we examine in detail below.

Stepping back for a moment, we emphasize that all TiRs can be understood to “represent” time, but this representation is *not* in units of time. The representation results either (i) from the integration of gestural/sensory system forces (non-autonomous TiRs), (ii) from a constant growth rate/frequency (autonomous TiRs) understood to be integration of surroundings forces, or (iii) from a combination of surroundings forces and forces from other TiRs (as in the case of coupled oscillators). Thus the systems we hypothesize represent time indirectly and imperfectly, in units of experienced force.

The utility of TiRs lies partly in their ability to indirectly represent time and partly in their ability to act on gestures or other systems. Table 1 below summarizes the types of TiRs discussed above. All TiRs are associated with a parameter vector τ that specifies the activation states at which the TiR acts upon other systems, along with a parameter vector χ whose sign determines whether actions open or close gestural gating systems. Autonomous TiRs are associated with a parameter ω which is either a growth rate (aperiodic TiRs) or angular frequency (periodic TiRs). The latter are also associated with a phase-coupling matrix. Non-autonomous TiRs are associated with a vector α of integration factors, which determines how input forces contribute to the growth of activation. Additional simulation parameters and details are described in Supplementary Material.

**TABLE 1:** Summary of TiRs.

Symbols	Autonomous/non-autonomous	Feedback source	Sub-classes	Periodic/aperiodic	Parameters
ε	Autonomous			Aperiodic	ω, χ/τ
θ	Autonomous			Periodic	ω, χ/τ, Φ
$\bar{T}$	Non-autonomous	CNS-external	Extra-gestural		α, χ/τ
$\hat{T}$	Non-autonomous	CNS-internal	Inter-gestural		α, χ/τ
$\tilde{T}$	Non-autonomous	G-internal	Inter-gestural		α, χ/τ

The motivations for including the different types of TiRs defined above relate to the goal of generating various empirical phenomena, which are described more specifically in Section “A hybrid model of gestural timing and speech rate control.” Broadly speaking, inter-gestural and extra-gestural non-autonomous TiRs are intended to provide mechanisms for control that involve internal and external feedback, respectively (see Section “Gestural systems and temporal control of gestural activation”). Autonomous periodic TiRs (coupled oscillators) provide precise control over the relative timing of movements, allowing the model to generate symmetric displacement patterns. Autonomous aperiodic TiRs allow the model to initiate and terminate a sequence of actions; as we develop in Section “A hybrid model of gestural timing and speech rate control,” these can be used to implement competitive selection, which is a sequencing mechanism.

### Deterministic behavior of time-representers and effects of stochastic forces

In order to better understand the behavior of TiRs, it is important to examine the covariance patterns of timing intervals that are generated by them. The analysis of covariance in temporal intervals is a basic tool for drawing inferences about the organization of temporal control in general (Wing and Kristofferson, 1973; Vorberg and Wing, 1996), and for articulatory timing in particular (Shaw et al., 2009, 2011; Tilsen, 2017). In order for interesting covariance patterns to arise, sources of stochastic variation must be present in the system. This section first establishes the deterministic, non-stochastic properties of temporal intervals in the current framework, and then examines how those temporal intervals covary in the presence of stochastic forces.

Under certain conditions, the time δ when a TiR acts on some other system (δ is relative to when TiR activation began to grow) is fully determined by its parameters. In the case of autonomous, aperiodic TiRs, the growth rate ω and action threshold τ determine δ. In two-dimensional ω/τ parameter space, constant δ are straight lines of positive slope, since increases of ω (which shorten δ) can be offset by increases of τ (which lengthen δ). Thus either changes in TiR rate ω or in its action threshold τ, or in some combination of the two, can generate the same change in action timing. This holds for τ and the integration rate α of non-autonomous TiRs as well, as long as the input force to the TiR is constant. For coupled oscillator TiRs, δ depends in complicated ways on the initial phases of the systems, the oscillator frequencies, and the strengths of phase coupling forces (putting aside oscillator amplitude dynamics).

For even a simple system of three gestures, there is a rich set of possible ways in which temporal control can be organized. How can the organization of control be inferred from empirical observations? What we call “noise” may be quite useful in this regard. An essential characteristic of natural speech is that it is unavoidably stochastic, and as a consequence, no two utterances are identical. We interpret stochastic forces here as variation across utterances in the influence of the surroundings on time-representing systems. Moreover, in modeling noise we distinguish between *global noise*—stochastic variation that affects all TiRs equally—and *local noise*—stochastic variation that differentially affects TiRs. This distinction is important because the relative amplitudes of local and global noise can influence timing patterns.

**FIGURE 8:**
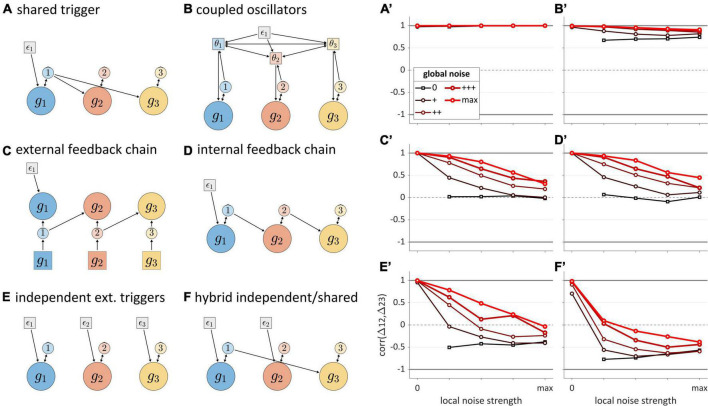
Noise-related correlation patterns for a variety of three-gesture systems. Panels **(A–F)** show model schemas and corresponding panels **(A’–F’)** show correlations of intervals between initiation of gestural systems. Local noise levels increase along the horizontal axes, while global noise levels are indicated by the lines in each panel. Cases where both global and local noise are zero are excluded.

The analysis of stochastic variation below focuses on correlations of successive time intervals between gestural initiations in three-gesture systems. These intervals are referred to as Δ12 and Δ23. We examine correlations (henceforth “Δ-correlations”) rather than interval durations, because correlations more directly reflect interactions between systems. Five different local and global noise levels were crossed, from 0 to a maximum level (see Supplementary Material: Simulations for further detail). Figures 8A–F show the structures of each model tested, and corresponding panels in Figures 8A’–F’ show how Δ-correlation varies as a function of global and local noise levels. Each line corresponds to a fixed level of global noise, and horizontal values of points represent different local noise levels.

The “shared trigger” model (Figure 8A) shows that if both non-initial gestures are activated by feedback from the initial one, Δ-correlation is trivially equal to 1, regardless of noise. The reason for this is simply that the same TiR (here $\hat{1}$) activates *g*_2_ and *g*_3_. Note that this trivial correlation occurs for external feedback control as well (not shown). The coupled oscillators model (Figure 8B) is unique among the systems examined in that it always produces non-trivial positive correlations. The reason for this has to do with phase coupling. Even when oscillator frequencies are heterogenous due to local noise, phase-coupling forces stabilize the oscillators at a common frequency. As long as phase-coupling forces are strong, local noise has relatively small effects on the phase evolution of oscillators. Global frequency noise always leads to positive correlations because it results in simulation-to-simulation variation in frequency that equally influences Δ12 and Δ23, causing them to covary positively. However, a more complex analysis of correlation structure in the coupled oscillators model in Tilsen (2017) has shown that when coupling strengths are also subject to noise, the model can generate negative correlations.

The external and internal feedback “chain models” (Figures 8C,D) exhibit nearly identical, complex patterns of correlation that depend on the relative levels of global and local noise. The patterns are nearly identical because the two models are topologically similar—they are causal chains—differing only in regard to the temporal delay associated with external sensory feedback. When there is no local noise, these chain models exhibit Δ-correlations of 1, since the global noise has identical effects on Δ12 and Δ23. Conversely, when there is no global noise, Δ-correlation is 0, since local noise has independent effects on Δ12 and Δ23. In-between those extremes, the correlation depends on the relative levels of local and global noise: increasing local relative to global noise leads to decorrelation of the intervals.

Unlike the other models, the independent extra-gestural triggers model (Figure 8E) and hybrid model (Figure 8F) can generate substantial negative correlations. In particular, negative correlations arise when *g*_2_ is influenced by local noise. This occurs because whenever the TiR which activates *g*_2_ does so relatively early or late, Δ12 and Δ23 will be influenced in opposite ways. Note that the negative correlations are stronger when the activation of *g*_1_ and *g*_3_ are caused by the same TiR, as is the case for the hybrid model (Figure 8F). At the same time, global noise induces positive Δ-correlation, counteracting the negative correlating effect of local noise. When we examine speech rate variation below, we will see that the opposing effects of global and local noise are not specific to “noise” *per se*: any source of variation which has similar effects on all TiRs tends to generate positive interval correlations, while the absence of such variation can lead to zero or negative correlation.

## A hybrid model of gestural timing and speech rate control

Equipped with a new logic of temporal control, we now develop a hybrid model of gestural timing which is designed to accommodate a wide range of empirical phenomena. The primary requirement of the model is that for each gesture which is hypothesized to drive articulatory movement in an utterance, the model must generate commands to activate and deactivate that gesture.

### Model space and hypotheses

For even a single CVC syllable, the set of all logically possible models is very large. Nonetheless, there are a number of empirical and conceptual arguments that we make to greatly restrict this space. Below we consider various ways in which gestural activation might be controlled for a CVC syllable uttered in isolation. Note that we adopt the modern “split-gesture” analysis in which constriction formation and constriction release are driven by separate gestural systems; this analysis has been discussed and empirically motivated in Nam (2007) and Tilsen, 2011, 2017. With that in mind we use the following gestural labeling conventions: C/c and R/r correspond to constriction formation and release gestures, respectively; upper case labels C/R correspond to pre-vocalic gestures (or, gestures associated with syllable onsets); lower case labels c/r correspond to post-vocalic gestures (or, gestures associated with syllable codas); and gestures/gesture pairs are subscripted according to the order in which they are initiated.

**FIGURE 9:**
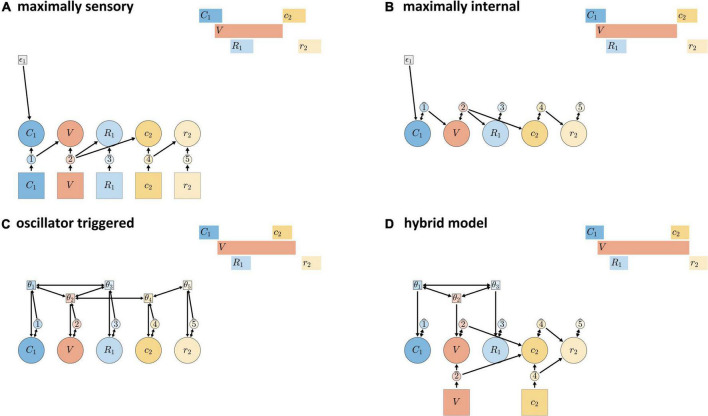
Candidate models of CVC syllables. **(A)** Maximally sensory model where all activation and deactivation is controlled by external sensory feedback. **(B)** Maximally internal model where all control is governed by internal feedback. **(C)** Fully oscillator-triggered model where all gestures are initiated by oscillators. **(D)** Hybrid model in which pre-vocalic gestural activation is oscillator-governed while post-vocalic activation is governed by either internal or external feedback.

The schemas in Figures 9A–C show “extreme” models that—though logically possible—are conceptually and empirically problematic. Figure 9A shows a “maximally sensory” model, where all gestural activation/deactivation is controlled by external feedback systems. This model is problematic because the time delay between efferent motor signals and afferent feedback is too long to be useful for some relative timing patterns, such as the relative timing of consonantal constriction and release in normal speech. Figure 9B shows a “maximally internal” model, where all gestural activation and deactivation is induced by inter-gestural TiRs (keeping in mind that initiation of activation of the first gesture in an utterance is always external). The maximally internal model is problematic because it has no way of allowing for external/sensory feedback to influence timing.

Schema (Figure 9C) shows an “oscillator triggered” model, where all gestures are activated by coupled oscillators. Under standard assumptions, this model is problematic because it cannot generate some empirically observed combinations of pre-vocalic and post-vocalic consonantal timing, as discussed in Tilsen (2018). For example, in a CVC syllable, the temporal intervals between the initiation of the vocalic gesture and the initiations of onset and coda consonantal gestures cannot be produced by a system of oscillators that govern all three of these events, given certain constraints on oscillator frequency, triggering, and coupling. The “standard” assumptions are: (i) that all oscillators have (approximately) the same frequency; (ii) that all oscillators trigger gestural initiation at the same phase of their cycle; and (iii) that only in-phase and anti-phase coupling are allowed. With these constraints, the model cannot generate empirically common combinations of pre-vocalic and post-vocalic temporal intervals, where prevocalic CV intervals are generally in the range of 50–100 ms (Tilsen, 2017) and post-vocalic VC intervals—periods of time from V initiation to post-vocalic C initiation—are in the range of 150–400 ms. Moreover, relaxing any of the three assumptions may be undesirable. Allowing oscillators to have substantially different frequencies can lead to instability and chaotic dynamics, unless coupling forces are made very strong. Allowing oscillators to trigger gestures at arbitrary phases is inconsistent with the neurophysiological interpretation: presumably one particular phase of the cycle represents maximal population spike rate and should be associated with the strongest triggering force. Allowing for arbitrary relative phase coupling targets, such as a relative phase equilibrium of 3π/2, may not be well-motivated from a behavioral or neurophysiological perspective.

Although the relatively extreme/monolithic models of Figures 9A–C are individually problematic, the mechanisms that they employ are practically indispensable for a comprehensive understanding of timing control. The hybrid control model (Figure 9D) is hypothesized to represent temporal control in typical adult speech. The model is described as “hybrid” because it uses coordinative/oscillator-based control for pre-vocalic timing, while allowing for internal or external feedback control for vocalic and post-vocalic timing. The model can be viewed as the combination of the following two more specific hypotheses:

*Pre-vocalic coordinative control hypothesis.* Control of the initiation of pre-vocalic consonantal constriction formation (C), release (R), and vocalic (V) gestures is governed by a system of coupled oscillators.

*Vocalic*/*post-vocalic feedback control hypothesis.* The deactivation of vowel gestures and the activation/deactivation of post-vocalic constriction (c) and release (r) gestures is governed by either internal or external feedback.

Below, we explain how each component of the model is motivated by a specific set of empirical phenomena.

#### Empirical motivation for pre-vocalic oscillator-based control

A major rationale for oscillator-triggered control is the phenomenon of symmetric displacement patterns (Tilsen, 2017, 2018). Such patterns were first described as the “c-center effect” in syllables with complex onsets (Browman and Goldstein, 1988). For a syllable with the form C1C2V, studies from a variety of languages have observed that the movements associated with the formation of the C1 constriction precede the movement associated with the vocalic posture, while the movements associated with the C2 constriction follow the movements associated with the vocalic posture; the C1 and C2 movement initiations tend to be approximately equally displaced in opposite directions in time from the initiation of the vocalic movement (Sproat and Fujimura, 1993; Byrd, 1995, 1996; Honorof and Browman, 1995; Kuhnert et al., 2006; Goldstein et al., 2007; Marin and Pouplier, 2010; Hermes et al., 2011, 2013; Tilsen et al., 2012). The pattern is remarkable because the order in which articulatory movements are initiated in such forms deviates from the order of segments in linear symbolic representations. The understanding of the c-center effect was significantly generalized by Nam (2007) and Tilsen (2017), where it was shown that a similar pattern of temporal displacement applies to the formation and release of the consonantal constriction in simple CV syllables: the constriction formation and release are displaced in opposite directions in time from the initiation of the vocalic movement. The only mechanism that has been proposed to explain symmetric displacement patterns is one in which the initiations of the gestures are governed by a system of coupled oscillators. With a combination of repulsive phase coupling between the oscillators that trigger consonantal gestures and attractive phase coupling between consonantal and vocalic oscillators, such a system naturally evolves toward a steady-state in which consonantal oscillator phases are displaced in opposite directions from the vocalic oscillator phase. Although the existence of symmetric displacement timing patterns does not prove that oscillators govern gestural timing, it is important to recognize that there exist no alternative models of these pervasive patterns.

A more indirect motivation for oscillator-triggered control comes from the observation that in the babbling stage of speech development, children employ an oscillatory cycle of jaw opening and closing to bootstrap the acquisition of CV syllables (MacNeilage et al., 1997; MacNeilage and Davis, 2000; Oller, 2000; Iverson et al., 2007). Furthermore, several studies have reported a coincidence of rhythmic activities in speech and non-speech domains (Thelen, 1979; Eilers et al., 1993; Iverson et al., 2007). It was argued in Tilsen (2014) that the oscillatory character of babble and its relation to oscillatory behaviors in non-speech actions suggest that oscillatory systems control the initiation of articulatory movements in CV forms.

#### Empirical motivation for vocalic/post-vocalic external feedback control

The primary motivation for incorporating external feedback control systems in the model is the common observation that word durations are lengthened in the presence of feedback perturbations (Houde and Jordan, 1998; Larson et al., 2001; Purcell and Munhall, 2006; Villacorta et al., 2007; Tourville et al., 2008; Cai et al., 2011). Such durational changes occur when auditory feedback is naturally or artificially degraded, and this occurs even in laboratory studies in which speakers are not accommodating listeners; the effect is known to be at least partly involuntary (Garnier et al., 2010; Zollinger and Brumm, 2011; Luo et al., 2018). It follows that there must be some temporal control mechanism that is responsible for increases in word duration in the absence of sensory feedback. The external feedback systems hypothesized to control the timing of vocalic/post-vocalic gestures are a minimal expansion of the model and are necessary for modeling the temporal effects of feedback perturbations.

Furthermore, recent evidence indicates that the temporal effects of auditory feedback perturbations are specific to vocalic/post-vocalic timing. The study in Oschkinat and Hoole (2020) found that post-vocalic intervals respond to temporal perturbations of feedback and that pre-vocalic intervals do not; specifically, subtle temporal delays of feedback imposed during a complex onset did not induce compensatory timing adjustments, while the same perturbations applied during a complex coda did. Another recent study (Karlin et al., 2021) found that temporal perturbations induced compensatory adjustments of vowel duration but not of onset consonant duration. Although the hybrid character of the model is a complication compared to purely feedforward or feedback control structure, it seems necessary to account for the dissociation in feedback sensitivity that was observed by these studies.

Moreover, there are a host of more indirect reasons for dissociating pre-vocalic and vocalic/post-vocalic control mechanisms. These are discussed in depth in Tilsen (2016) but are briefly re-iterated here. First, the coarticulatory patterns exhibited by young children differ substantially between pre-vocalic and post-vocalic contexts: children show hyper-coarticulatory patterns between CV but hypo-coarticulatory patterns between VC (Kent, 1983; Hawkins, 1984; Repp, 1986; Goodell and Studdert-Kennedy, 1993; Sussman et al., 1999; Katz and Bharadwaj, 2001). Second, the patterns of sequencing errors exhibited by children in the early word stage are highly asymmetric for onsets and codas [see section 3.2 of Tilsen (2016) for a comprehensive analysis]. Third, a unified understanding of several forms of typological variation in syllable structure is made possible by hypothesizing pre-/post-vocalic asymmetries in the use of feedback for temporal control (Tilsen, 2016).

#### Empirical motivation for internal feedback control

The primary motivation for including internal feedback control systems in addition to external ones is the observation that temporal control is possible under circumstances in which external feedback is not available, for example during loud cocktail parties, for speakers with complete hearing loss, or during subvocal rehearsal (internal speech) with no articulatory movement. Thus in order for a model of temporal control to be empirically adequate, it is necessary to include internal feedback systems. There is a wide range of argumentation and evidence for the use of internal feedback control of movement, both generally (Miall and Wolpert, 1996; Kawato and Wolpert, 1998; Kawato, 1999; Thoroughman and Shadmehr, 1999; Shadmehr and Krakauer, 2008) and specifically in speech motor control (Guenther and Perkell, 2004; Max et al., 2004; Hickok, 2012; Guenther and Hickok, 2016; Parrell et al., 2018). Moreover, internal feedback systems are incorporated in a variety of speech production models (Tourville and Guenther, 2011; Hickok, 2012; Guenther and Hickok, 2016). However, most of the studies providing evidence for internal feedback control focus on the control of movement *via* predictive state estimation and error correction. These functions are instances of control *from* intentions, rather than control *of* (the timing of) intentions.

The inclusion of internal TiRs for control of timing in the hypothesized model follows from the reasoning that, in the absence of external feedback, some mechanism is needed to govern timing. For the reasons discussed above, this mechanism cannot be oscillator-based control. Because internal feedback systems are already motivated by their role in predictive state estimation and error correction, they are a natural candidate for a parsimonious model of timing control.

### External influences on parameters

Here and following sections, some specific predictions of the hypotheses are examined. A key point about the model is that parameters of TiRs are context-dependent: they vary in ways that are conditioned on factors associated with TiR system surroundings, so-called “external factors.” Here we demonstrate two ways in which external factors may influence timing. An innovation of the model is the idea that these factors can have differential influences on external vs. internal TiR parameters.

**FIGURE 10:**
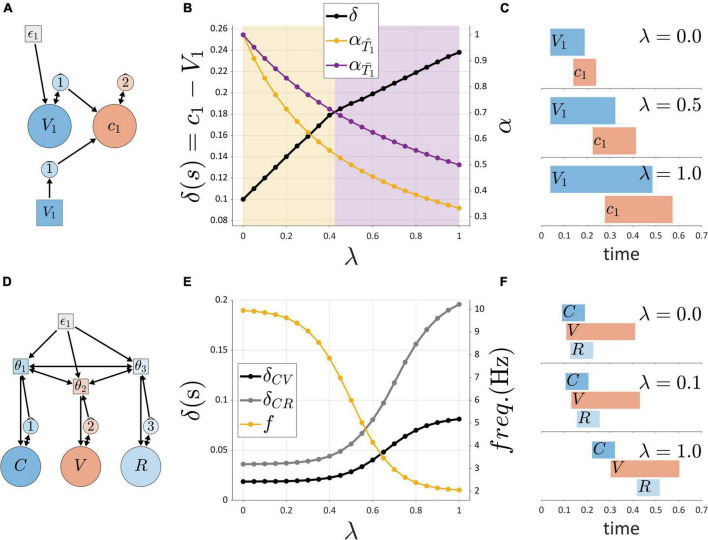
Simulations of external influences on parameters. **(A)** Schema for post-vocalic control with both internal and external TiRs. **(B)** Dual axis plot showing how δ (left side) and integration rates α (right side) change with self-attention parameter λ. **(C)** Gestural activation intervals for several values of λ. **(D)** Model schema of pre-vocalic coordinative control. **(E)** Dual axis plot showing effect of rate parameter λ on δ-values (left side) and frequencies (right side). **(F)** Gestural activation intervals for several values of λ.

Figures 10A–C demonstrate the effects of variation in a hypothetical contextual factor of *self-attention*, or “attention to one’s own speech,” which is represented by a variable, λ. The self-attention variable λ ranges from 0 to 1, where 0 represents minimal attention to one’s own speech, and 1 represents maximal attention. The figure summarizes simulations of the system shown in Figure 10A, where activation of a post-vocalic constriction gesture *c*_1_ is potentially caused by an internal or external TiR representing feedback from the vocalic gesture V_1_. This is the hypothesized organization of post-vocalic control in the hybrid model. By hypothesis, the force integration rates of internal and external TiRs are differentially modulated by self-attention λ, such that α = α′/(1 + βλ), where β_internal_ < β_external_. This reflects the intuition that when one attends to feedback more closely, feedback-accumulation (i.e., force-integration) rates of TiR systems are diminished, so that TiRs take longer to act on gestures. This diminishing effect applies more strongly to internal feedback than external feedback. As a consequence, there is a value of λ such that as λ is increased, initiation of *g*_2_ switches from being governed by the internal TiR to the external one. In the example, the transition occurs around λ = 0.425, where a change is visible in the slope relating the control parameter λ and the interval δ (the time between initiation of *V*_1_ and *c*_1_). Gestural activation intervals associated with three values of λ are shown in Figure 10C.

Figure 10B shows that when TiR parameters are differentially modulated by an external influence, transitions between internal and external feedback control can occur. In the above example, the external influence was posited to represent “self-attention” and its state was encoded in the variable λ. This variable was then hypothesized to differentially adjust external vs. internal non-autonomous TiR growth rates. Another way in which the same effect can be derived is by allowing the external variable λ to differentially adjust TiR action-thresholds.

Another parameter that can respond to external factors is the frequency of the coupled oscillators which are hypothesized to govern prevocalic gestural initiation, as in Figure 10D. Suppose that the external factor here is a mechanism that controls oscillator frequency *via* an external variable called “pace.” As with self-attention, the external variable of pace ranges from 0 to 1, with 0 corresponding to minimal pace and 1 corresponding to maximal pace. However, because of the frequency constraint hypothesis, we cannot simply allow the oscillator frequencies to respond linearly to changes in pace. Instead, we impose soft upper and lower frequency bounds by attenuating the effect of the pace parameter λ on frequency *f*. This is accomplished by making the effective frequency a non-linear function of λ, as shown in Figure 10E (right side) and Figure 10F. The consequence of this limitation on *f* is that intervals which are governed by coordinative control are predicted to exhibit non-linear responses to variation in the external factor: here we can see that the δ_*CV*_ and δ_*CR*_ plateau at extreme values of λ.

In the section “A model of speech rate control with selectional effects,” we combine the above effects of self-attention and pace into a general model of the control of speech rate. But first we introduce another important mechanism, which allows the model to organize the subsystems of larger utterances.

### Parallel domains of competitive selection

Competitive selection (or competitive queuing) is a dynamical mechanism that, given some number of actions, iteratively selects one action while preventing the others from being selected. The concept of competitive selection of actions originates from Grossberg (1987), and many variations of the idea have been explored subsequently, both within and outside of speech (Bullock and Rhodes, 2002; Bullock, 2004; Bohland et al., 2010; Bhutani et al., 2013; Tilsen, 2013; Glasspool, 2014; Kristan, 2014). One of the key ideas behind the mechanism is that a serial order of actions is encoded in an initial activation gradient, such that prior to the performance of an action sequence, the first action in the sequence will have the highest relative activation, the second action will have the next highest activation, and so on. The growth of activation is a “competition” of systems to be selected, and selection is achieved by reaching an activation threshold. Moreover, action selection is mutually exclusive, such that only one action can be selected at a time.

**FIGURE 11:**
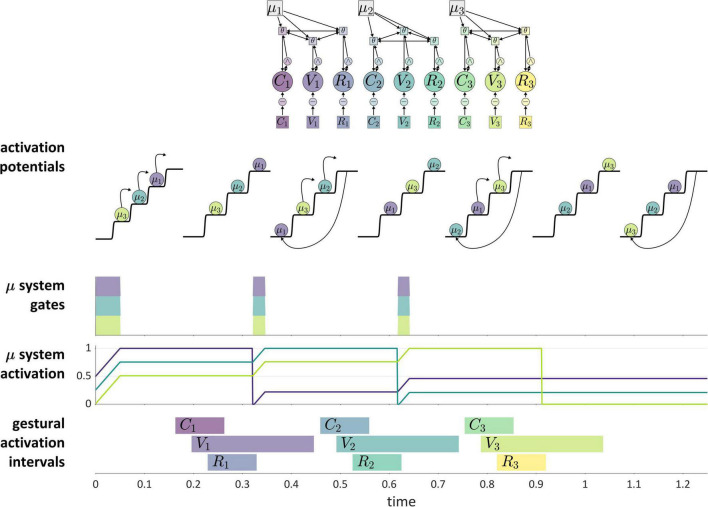
Illustration of competitive selection for a sequence of three CV syllables. **(Top)** Model schema. Activation potentials with arrows show transitions between states, and potentials without arrows shown quasi-steady states. μ-gating system states are shown (shaded intervals are open states). **(Bottom)** Gestural activation intervals.

Figure 11 shows how these ideas are understood in the current model. The “actions” which are competitively selected in this example are three CV syllables, and the selection of these actions is governed by systems that we refer to as *μ-systems*. As shown in the model schema, each μ-system de-gates a system of coupled oscillators, which in turn activate gestures. Each of the μ-systems is associated with a μ-gating system that—when open—allows the corresponding μ-system activation to grow. Notice that at time 0 (before the production of the sequence), the pattern of relative activation of μ-systems corresponds to the order in which they are selected. When μ-system gates are open, μ-system activations grow until one of the systems reaches the selection threshold. At this point, all μ-gating systems are closed, which halts growth of μ-system activation. The selected μ-system is eventually suppressed (its activation is reset to 0) by feedback—specifically by the inter-gestural TiR associated with the last gesture of the syllable, in this case the vowel gesture. This causes all μ-systems to be de-gated, allowing their activations to grow until the next most highly active μ-system reaches the selection threshold. This three-step process—(i) de-gating and competition, (ii) selection and gating of competitors, and (iii) feedback-induced suppression of the selected system—iterates until all of the μ-systems have been selected and suppressed. See Supplementary Material: Model details for further information regarding the implementation.

A more abstract depiction of a competitive selection trajectory is included in the activation potentials of Figure 11. The potentials without arrows are relatively long epochs of time in which μ-systems exhibit an approximately steady-state pattern of activation. The potentials with arrows correspond to abrupt intervening transitions in which the relative activation of systems is re-organized by the competitive selection/suppression mechanism. Along these lines, the dynamics of competitive selection have been conceptualized in terms of operations on discrete states in Tilsen, 2019a,c.

There are two important questions to consider regarding the application of a competitive selection mechanism to speech. First, exactly what is responsible for suppressing the currently selected μ-system? In the example above, which involves only CV-sized sets of gestures, it was the internal TiR associated with the last gesture of each set. Yet a more general principle is desirable. Second, what generalizations can we make about the gestural composition of μ-systems? In other words, how is control of gestural selection organized, such that some gestures are selected together (*co-selected*) and coordinatively controlled, while others are competitively selected *via* feedback mechanisms? This question has been discussed extensively in the context of the Selection-coordination theory of speech production (Tilsen, 2014, 2016), where it is hypothesized that the organization of control follows a typical developmental progression. In this progression, the use of external sensory feedback for suppression/de-gating is replaced with the use of internal feedback, a process called *internalization of control*.

The are two important points to make about internalization. First, internalization of control is partly optional, resulting in various patterns of cross-linguistic and inter-speaker variation which are detailed in Tilsen (2016) and which we briefly discuss in the section “No direct control of the timing of target achievement.” Second, internalization is flexible within and across utterances, such that various contextual factors (e.g., self-attention) can influence whether external or internal feedback TiRs are responsible for suppressing selected μ-systems.

Furthermore, a recently developed theory of syntactic organization in speech (Tilsen, 2019c) argues that there are two interacting domains of competitive selection. This is known as the *parallel domains hypothesis*. One of these domains involves “gestural-motoric” organization of the sort illustrated above, where gestures are organized into competitively selected sets (μ-systems). The other involves “conceptual-syntactic” organization in which concept systems are organized into competitively selected sets. The hypotheses advanced in Tilsen (2019c) hold that sets of co-selected conceptual systems correspond loosely to the prosodic unit called the *phonological word* (a.k.a. p-wrd, or ω), which has the property that there is a single accentual gesture associated with set of co-selected conceptual systems. Moreover, under normal circumstances speakers do not interrupt (for example by pausing) the gestural competitive selection processes which are induced by selection of a phonological word.

**FIGURE 12:**
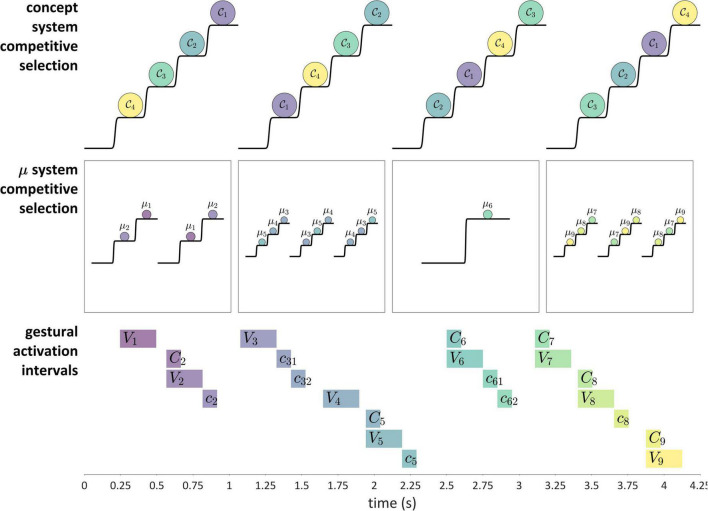
Illustration of parallel domains of competitive selection for an utterance with the structure. **(Top)** Concept systems C are competitive selected. **(Middle)** Selection a concept system de-gates corresponding μ-systems which themselves are competitively selected. **(Bottom)** Gestural activation intervals generated by the model.

These parallel domains of conceptual-syntactic and gestural-motoric competitive selection are illustrated Figure 12 for an utterance which would typically be analyzed as four prosodic words, such as [*a dog*] [*and a cat*] [*chased*] [*the monkey*]. Note that to conserve visual space release gestures have been excluded. The top panel shows the sequence of epochs in competitive selection of concept systems 𝒞. Each of these could in general be composed of a number of co-selected subsystems (not shown). For each epoch of concept system selection, there is a corresponding series of one or more epochs of competitive selection of gestural systems. The model accomplishes this by allowing the concept systems to de-gate the corresponding sets of μ-systems. Within each of these sets of μ-systems, the appropriate initial activation gradient is imposed. Further detail on the implementation is provided in the Supplementary Material.

Although there is no *a priori* constraint on the number of domains of competitive selection that might be modeled, the parallel domains hypothesis that we adopt makes the strong claim that only two levels are needed—one for conceptual-syntactic organization and one for gestural-motoric organization. We examine some of the important consequences of these ideas in Section “Reinterpretation of prosodic phrase structure and boundaries,” regarding phrasal organization. One aspect of prosodic organization which we do not elaborate on specifically in this manuscript involves the metrical (stress-related) organization of gestures, but see Tilsen (2019b) for the idea that the property of “stress” relates to which sets of co-selected gestures (μ-systems) may include accentual gestures, which in turn are responsible for transient increases in self-attention.

### A model of speech rate control with selectional effects

When given verbal instructions to “talk fast” or “talk slow,” speakers are able to produce speech that listeners can readily judge to be relatively fast or slow. To quantify this sort of variation in tempo, speech rate is often measured as a count of events per unit time, e.g., syllables per second or phones per second. There are several important points to consider about these event-rate quantities, which call into question whether speakers control tempo as a rate of events, *per se*. First, in order to be practically useful, an event rate must be measured over a period of time in which multiple events occur. Hence event rates are unlikely to be controlled instantaneously, since such measures cannot be robustly defined in a moment-to-moment fashion. Second, there is no consensus on which events are the appropriate ones to count—phones, syllables, words, or something else? In the current framework, many commonly used units do not even have an ontological status. In order for an event rate to be controlled, it stands to reason that the relevant events should have some degree of cognitive reality. Third, even if we ignore the above problems, there is no evidence to my knowledge that speakers directly control rate quantities such as syllables/second or phones/second. Hence there is reason to doubt that the quantity which speakers attempt to control should be conceptualized as a rate of events. If speakers do not in fact control speech rate as an event rate *per se*, then what are speakers controlling in order to speak fast or slow?

The *attentional modulation hypothesis* (Tilsen, 2018) holds that speakers control rate by modulating their attention to feedback of their own speech (*self-attention*), and specifically do so in a way that, as self-attention increases, prioritizes external/sensory feedback over internal feedback. Furthermore, along with modulating self-attention, speakers may adjust pacing, that is, the frequencies of gestural planning oscillators. The separate effects of varying these external factors were already demonstrated in Section “External influences on parameters.”

In addition, a mechanism is needed to account for the phenomenon of boundary-related lengthening. Many empirical studies have shown that speech slows down as speakers approach the ends of phrases, with greater slowing and increased likelihood of pausing statistically associated with “higher-level” phrase boundaries (Byrd and Saltzman, 1998, 2003; Byrd, 2000; Byrd et al., 2006; Turk and Shattuck-Hufnagel, 2007, 2020b; Krivokapić, 2014). One approach to understanding the mechanism responsible for such effects is the π-gesture model of Byrd and Saltzman (2003), in which it was hypothesized that boundary-related lengthening is caused by a special type of clock modulating system, a “π-gesture.” This clock-modulating system, when active, slows down the rate of a hypothesized nervous system-internal global clock, relative to real time. Gestural activation dynamics evolve in the internal clock coordinate, and so gestural activation intervals are extended in time when a π-gesture is active. Furthermore, it was suggested in Byrd and Saltzman (2003) that the degree of activation of a π-gesture varies in relation to the strengths of prosodic boundaries, such that stronger/higher-level boundaries are associated with greater π-gesture activation and hence more slowing.

How can the phenomenon of boundary-related lengthening be conceptualized in the current framework, where there is no global internal clock for gestural systems? A fairly straightforward solution is to recognize that in effect, each gestural system has its own “local clocks,” in the form of the internal and external feedback TiRs, whose integration rates are modulated by self-attention. In that light, it is sensible to adapt the π-gesture mechanism by positing that self-attention effects on TiR parameters tend to be greater not only in the final set of gestures selected in each prosodic word (i.e., final μ-system), but also in the final set of co-selected conceptual systems (i.e., the final μ-system). As for why it is the final set of selected systems that induces these effects, we reason that speakers may attend to sensory feedback to a greater degree when there are fewer systems that remain to be selected. At the end of an utterance, there are no more systems that remain to be selected, and thus self-attention is greatest. We refer to this idea as the *selectional anticipation hypothesis*, because anticipation of upcoming selection events is proposed to distract a speaker from attention to feedback of their own speech. Although this hypothesis is admittedly a bit ad hoc, and alternative explanations should be considered, we show below that the implementation of this idea is sufficient to generate the lengthening that occurs at the ends of phrases.

**FIGURE 13:**
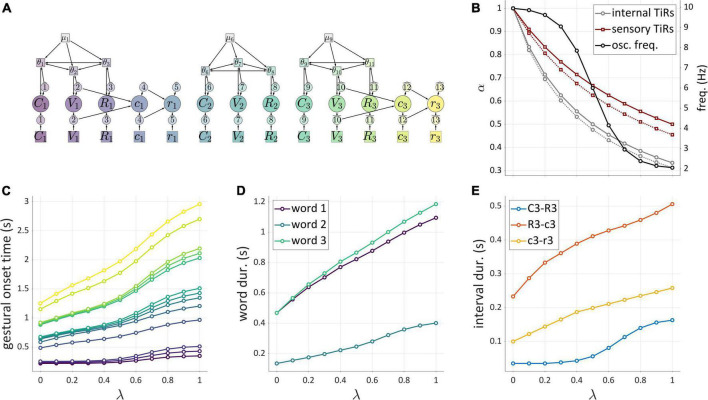
Simulation of variation in speech rate, as controlled by correlated chagnes in self-attention and pacing, both indexed by λ. **(A)** Model schema showing three syllables with the forms CVC, CV, and CVC. **(B)** Relations between λ and feedback TiR integration rates (α) and oscillator frequencies. **(C)** Times of gestural initiation for each value of λ simulated. **(D,E)** Word durations and interval durations of the third word.

Putting the above ideas together, Figure 13 shows how interval durations change as a function of attentional modulation. The utterance here is a competitively selected sequence of three syllables with forms CVC, CV, CVC, as shown in Figure 13A. Note that the organization of each syllable conforms to the hybrid control model, entailing that pre-vocalic timing is coordinative and vocalic/post-vocalic timing is feedback-based. As in the section “External influences on parameters,” the integration rates of external (sensory) and internal TiRs, along with oscillator frequencies, are made to vary in response to changes in a control parameter λ; these relations are shown in Figure 13B. In addition, the integration rate parameters associated with the final set of gestures are even more strongly modulated by λ (dotted lines of Figure 13B), to implement the selectional anticipation hypothesis; the consequences of this are evident in the contrast between word 1 and word 3 durations in Figure 13D. The initiation times of gestures for each of the 11 values of λ that were simulated are shown vertically in Figure 13C.

By simulating variation in speech rate, we are able to generate some of the most essential predictions of the hybrid control model, introduced in Section “Model space and hypotheses.” Recall that this model combined two hypotheses: prevocalic coordinative control and post-vocalic feedback-control. These hypotheses are associated with the following three predictions:

(i) *Prevocalic attenuation*. The prevocalic coordinative control hypothesis holds that initiation of the prevocalic constriction and release gestures, along with initiation of the vocalic gesture, is controlled by a system of coupled oscillators. Moreover, the frequency constraint hypothesis was shown in the section “External influences on parameters” to predict that intervals between these initiations attenuate as rate is increased or decreased. This effect can be seen in Figure 13E for the C_3_-R_3_ interval, which is the interval between constriction formation and release. In other words, the prediction is that prevocalic timing is only so compressible/expandible, no matter how quickly or slowly a speaker might choose to speak.
(ii) *Postvocalic expandability*. Conversely, the post-vocalic feedback-control hypothesis holds that there is a transition from internally to externally governed control, and that there should be no limits on the extent to which increasing self-attention can increase the corresponding interval durations. This prediction is shown in Figure 13E for the R_3_-c_3_ interval (which loosely corresponds to acoustic vowel duration) and the c_3_-r_3_ interval (related to constriction duration). These intervals continue to increase as attention to feedback is increased.
(iii) *Sensitivity to feedback perturbation*. Finally, a third prediction of the model is that, when external feedback governs post-vocalic control (as is predicted for slow rates), perturbations of sensory feedback will influence post-vocalic control but not prevocalic control.

How do these predictions fare in light of current evidence? The ideal tests of predictions (i) and (ii) require measurements of temporal intervals produced over a wide range of variation in global speech rate. Unfortunately, most studies of the effects of speech rate do not sufficiently probe extremal rates, since many studies use categorical adverbial instructions (e.g., *speak fast* vs. *speak normally* vs. *speak slowly*). One exception is a recent study using an elicitation paradigm in which the motion rate of a visual stimulus iconically cued variation in speech rate (Tilsen and Hermes, 2020). Utterance targets were words with either intervocalic singleton or geminate bilabial nasals (/ima/ and /imma/). The study observed that the timing of constriction formation and release of singleton /m/ exhibited a non-linear plateau at slow rates, similar to the prediction for the c_3_-r_3_ interval in Figure 13E. This is expected given the assumption that the formation and release gestures are organized in the onset of the second syllable of the target words. In contrast, the durations of constriction formation-to-release intervals of geminate /mm/ did not attenuate: they continued to increase as rate slowed. This is expected if the initiation of the geminate bilabial closure is associated with the first syllable and its release with the second. Although the dissociation of effects of rate on singletons vs. geminates is not the most direct test of the hybrid model hypothesis, it shows that more direct tests are warranted.

Regarding prediction (iii), a recent study has indeed found evidence that post-vocalic intervals respond to temporal perturbations of feedback and that pre-vocalic intervals do not (Oschkinat and Hoole, 2020). This study found that subtle temporal delays of feedback imposed during a complex onset did not induce compensatory timing adjustments, while the same perturbations applied during a complex coda did. This dissociation in feedback sensitivity is a basic prediction of the hybrid model. Another recent study (Karlin et al., 2021) has found that temporal perturbations induced compensatory adjustments of vowel duration but not of onset consonant duration (codas were not examined). There may be other reasons why temporal feedback perturbations have differential effects on prevocalic and vocalic/post-vocalic intervals, and certainly there is much more to explore with this promising experimental paradigm. Nonetheless, effects that have been observed so far are remarkably consistent with the predictions of the hybrid control model.

## General discussion

The informal logic developed here has many consequences for phonological theories. Below we discuss an important point about control of target timing along with two of the most important consequences of the model. First, the framework does not allow for direct control over the timing of articulatory target achievement, and we will argue that this is both conceptually desirable and empirically consistent. Second, structural entities such as syllables and moras can be re-interpreted in relation to differences in the organization of control. Third, there is no need to posit the existence of different types of phrases, nor a hierarchical organization of phrases: the appearance of prosodic “structure” above the phonological word can reinterpreted more simply as variation in self-attention conditioned on selection of prosodic words.

### No direct control of the timing of target achievement

Some researchers in the AP/TD framework have explicitly hypothesized that control of timing of target achievement is a basic function available in speech (Gafos, 2002), or have implicitly assumed such control to be available (Shaw et al., 2011). More generally, outside of the AP/TD framework, it has been argued that speakers prioritize control of the timing of articulatory and acoustic target events over control of the initiation of the very same actions that are responsible for achieving those targets (Turk and Shattuck-Hufnagel, 2014, 2020a,2020b). “Target achievement” is defined here as an event in which the state of the vocal tract reaches a putative target state that is associated with a gestural system.

Direct control of the timing of gestural target achievement is prohibited by our logic because TiRs control when gestural systems become active and cease to be active, and neither of these events fully determines the time at which targets are achieved. The TiR framework of course allows for *indirect* control of target achievement timing, *via* the trivial fact that target achievement depends in part on when a gesture is activated. Yet other factors, which are outside the scope of the TiR model, play a role as well. In standard TD (Saltzman and Munhall, 1989) these factors include the strengths of the forces that gestural systems exert on a tract variable systems—both driving forces and dissipative damping forces—as well as how these forces are blended when multiple gestural systems are active. Or, in an alternative model of how gestures influence tract variable control systems (Tilsen, 2019a), the relevant factors are the strengths, timecourses, and distributions of inhibitory and excitatory forces that gestural systems exert on spatial fields that encode targets. In either case, target achievement cannot be understood to be controlled directly by TiRs.

A major conceptual issue with direct control of the timing of target achievement is that it requires an unrealistically omniscient system that has accurate knowledge of the future. In order to control exactly when a target is achieved, a control system must initiate a movement at precisely the right time, which in turn requires that the system is able to anticipate the combined influences on the vocal tract state of all currently active subsystems and all subsystems which might become active in the near future. This all-knowing planner must accomplish these calculations before the critical time at which the movement must be initiated. While such calculations are not in principle impossible, they do require a system which has access to an implausibly high degree of information from many subsystems.

A primary empirical argument for direct control of target achievement is premised on the claim that there is less variability associated with timing of target achievement than variability associated with timing of movement onsets. This is argued in Turk and Shattuck-Hufnagel, 2014 to suggest that timing of target achievement is not only independently controlled, but also prioritized over timing of movement initiation. The difference in variability upon which the argument is premised has been observed in non-speech studies in which an actor must hit or catch a moving object. Yet these sorts of non-speech examples do not necessarily translate to speech, because in articulation there are no uncontrolled moving objects that the effectors must collide with at the right place in space and time—speech is simply not like catching a ball. Indeed, only one study of speech appears to have concluded that there is less variability in target vs. initiation timing (Perkell and Matthies, 1992), and this interpretation of the data is highly questionable due to differences in how the two events were measured.

Empirically observed phonetic and phonological patterns indeed provide the strongest argument *against* direct control of target achievement timing. Phonetic reduction of targets, which can arise from insufficient allotment of time for a target to be achieved, is rampant in speech. The “perfect memory” example of Browman and Goldstein (1990) shows how at fast speech rates the word-final [t] can be not only acoustically absent but also quite reduced kinematically when the preceding and following velar and bilabial closures overlap. If speakers prioritized the timing of the [t] target relative to either the preceding or following targets, this sort of reduction presumably would happen far less often. The prevalence of historical sound changes that appear to involve deletion of constriction targets argues against the notion that speakers are all that concerned with achieving targets. Certainly, the consequences of failing to achieve a target are usually not so severe: in order to recognize the intentions of speakers, listeners can use semantic/contextual information and acoustic cues that are not directly related to target achievement. Rather than being a priority, our informal logic views target achievement as an indirect and often not-so-necessary consequence of activating gestural systems.

### Reinterpretation of syllabic and moraic structure

Many phonological theories make use of certain entities—syllables (σ) and moras (μ)—as explanatory structures for phonological patterns. These entities are viewed structurally as groupings of segments, with moras being subconstituents of syllables, as was shown in Figure 2B. Selection-coordination theory (Tilsen, 2014, 2016) has argued that these entities, rather than being parts of a structure, should be thought of as different classes of phonological patterns that are learned in different stages of a particular developmental sequence, over which the organization of control changes. This idea is referred to as the *holographic hypothesis*, because it holds that what appears to be a multi-level structure of syllables and moras is in fact a projection over developmental time of two different forms of organization which do not exist simultaneously. This is loosely analogous to a hologram, which encodes a three-dimensional image in two dimensions.

**FIGURE 14:**
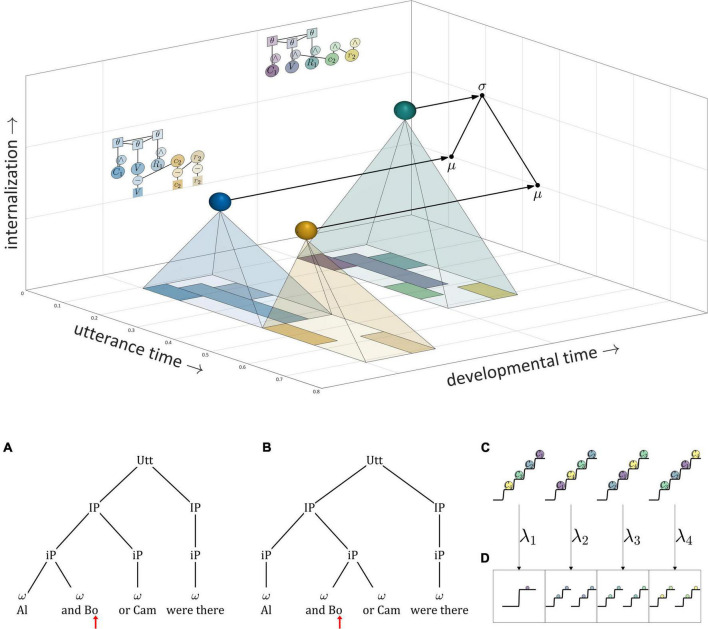
**(Top)** Visualization of the holographic hypothesis, for a CVC form. In an early stage of development, control over the post-vocalic constriction is based entirely on sensory feedback. Phonological patterns learned in this stage of development are described with moraic structure. In a later stage of development, control has been internalized, and phonological patterns learned in this stage are described with syllabic structure. **(Bottom)** Hierarchical prosodic structure reinterpreted as variation in attentional modulation of control parameters. **(A** vs. **B)** Alternative hierarchical prosodic structures purported to encode a difference in conceptual grouping. Red arrows indicate timepoint discussed in the text. **(C,D)** In different epochs of concept system selection, self-attention (λ) may differ, resulting in differences in temporal control.

The holographic hypothesis is exemplified in Figure 14 (top) for a CVC syllable. Early in development, the post-vocalic constriction gesture is controlled entirely by sensory feedback (i.e., extra-gestural TiRs), and so phonological patterns learned at this time are associated with a moraic structure, reflecting a stronger differentiation in control of pre-vocalic and post-vocalic articulation. Subsequently, speakers learn to activate and deactivate the post-vocalic constriction/release with internal TiRs, which is an instance of internalization. This leads to initiation of the post-vocalic constriction before termination of the vocalic gesture, and hence an increase in articulatory overlap/coarticulation. Phonological patterns learned in conjunction with this internalized organization of control are associated with syllables, rather than moras. Similar reasoning applies to other syllable shapes such as {C}{CV}→{CCV} and {CV}{V}→{CVV}, where developmental transitions in the internalization of control can account for cross-linguistic phonetic and phonological variation (Tilsen, 2016).

Exactly what causes internalization and governs its progression are open questions that presumably relate to information transmission. More internalization is associated with a greater rate of information production in speech, or in other words, increased efficiency of communication. Conversely, too much internalization can result in degrees of articulatory overlap which sacrifice perceptual recoverability (Liberman et al., 1967; Fowler and Rosenblum, 1991; Chitoran and Goldstein, 2006; Gick et al., 2006), reflecting constraints on channel capacity. It is far from clear how these opposing considerations—information rate vs. channel capacity—might be mechanistically manifested in a model of utterance-timescale processes. Informational aspects of speech, which by definition require analysis of the space of possible state trajectories of gestural systems, necessarily involve attention to patterns on lifespan timescales and speech-community spatial scales. Thus the challenge lies in understanding how these relatively large timescale informational forces translate to changes in utterance-scale control.

### Reinterpretation of prosodic phrase structure and boundaries

There are many prosodic theories in which prosodic words (ω) are understood to be hierarchically structured into various types of phrases. A “phrase” in this context simply refers to a grouping of prosodic words. Different types of phrases have been proposed, with two of the most popular being the “intonational phrase” (IP) and “intermediate phrase” (iP) from Beckman and Pierrehumbert (1986); these were shown in Figure 2B. Many theories additionally posit that these types of phrases can be recursively hierarchically organized (Ladd, 1986; Féry, 2010; Ito and Mester, 2013), such that a given type of phrase can contain instances of itself. In general, the motivations for positing phrase structures of this sort are diverse and too complex to address in detail here, but most of them relate either (i) to the likelihood that certain phonological patterns will occur in some portion of an utterance, or (ii) to statistical patterns in measures of pitch or duration observed in longer utterances.

To provide an example, consider the question: *Who was in the library?*, answered with the utterance *Al and Bo or Cam were there*. This response has two probable interpretations, and in many theories these would be disambiguated by the prosodic structures shown in Figure 14 (bottom: A vs. B):

The motivation for positing the structural distinction between Figure 14A and Figure 14B is that it can account for certain empirical patterns related to conceptual grouping. Consider specifically the period of time in the vicinity of the red arrows, near the end of the production of *Bo*, which is often conceptualized as a phrase “boundary.” Here utterance Figure 14A, compared to Figure 14B, will tend to exhibit a larger fall of pitch, greater boundary-related lengthening, and a greater likelihood of a pause. The pitch of the following word may also start at a higher value. Hierarchical structural analyses hold that these differences occur because there is a “higher-level boundary” at this location in Figure 14A than in Figure 14B, that is, an intermediate phrase boundary vs. a prosodic word boundary.

The logic of multilevel competitive selection makes hierarchical or recursive phrasal structure unnecessary. If anything, our framework corresponds to a flat, anarchical organization of prosodic words—though more appropriately it rejects the notion that prosodic words are parts of structures in the first place, and “boundaries” are seen as wholly metaphoric. How can regularities in intonational patterns such as in Figure 14A vs. Figure 14B be understood, without the notions of phrase hierarchies and boundaries?

Recall that each prosodic word is one set of co-selected concept systems, which are associated with some number of sets of co-selected gestural systems (Figure 11). Furthermore, recall that boundary-related lengthening was interpreted as a decrease in integration rates of feedback TiRs, and this parameter modulation is proposed to be greater for the last set of systems in a competitively selected set (the selectional anticipation hypothesis), as shown for the word durations in Figure 13D. This reasoning leads to an alternative understanding of why there exists phonetic and phonological variation that correlates with prosodic organization: rather than being due to “structural” differences, the variation arises from differences in how TiR parameters are modulated for each prosodic word, as suggested by the arrows in Figures 14C,D. Rather than constructing a structure of prosodic words for each utterance, speakers simply learn to adjust self-attention in a way that can reflect conceptual relations between systems of concepts. Presumably many forms of discourse-related and paralinguistic information can be signaled in this way, including focus phenomena such as emphatic and contrastive focus. In other words, to emphasize information for listeners, speakers simply emphasize that information for themselves.

## Conclusion

To conclude, we return to the initial questions of this manuscript: (i) what determines the duration of that *shush* that you gave to the loud person in the library, and (ii) how do you slow down the rant to your friend in the coffee shop? According to the feedback-based logic of temporal control, your *shush* duration is most likely determined by a sensory feedback-based control system (an external, non-autonomous TiR), and depending upon various factors (how angry you are, how far away the loud student is), you will diminish the integration rate of the TiR and/or increase its threshold to extend the duration of the sound. Later on in the coffee shop, you slow down your rant in effect by doing the same thing: increasing self-attention.

One possible criticism of the framework presented here is that it is too complex. While it is fair to assert that the model proposed here is complex compared to other models, this manuscript has shown that in all cases the complexity is warranted, in order to for the model to be empirically adequate. Simpler models are simply not able to generate the full range of temporal patterns which occur in speech. Given that empirically observed temporal patterns in speech are complicated, it is not surprising that the mechanisms used to generate speech must reflect that complexity.

There are several important conceptual and theoretical implications of our informal logic. First, all control of timing must be understood in terms of systems and their interactions, and this understanding involves the formulation of change rules to describe how system states evolve in time. Second, the systems which control timing do not “represent” time in any direct sense; the states of systems are defined in units of activation, and activation is never a direct reflection of elapsed time. Instead, it is more appropriate to say that timing is controlled *via* the integration of force, in combination with learned yet adjustable thresholds that determine when systems act. Third, the timing of target achievement is not a controlled event. Finally, much of the theoretical vocabulary that spans the range of timescales portrayed in Figure 2 is contestable, and new interpretations of empirical patterns can be derived from our logic. This applies to units such as syllables and moras, and also to hierarchical and recursive organizations of phrases. Ultimately the logic is useful because it facilitates a unified understanding of temporal patterns in speech, from the short timescale of articulatory timing to the large timescale of variation in speech rate.

## Data availability statement

The datasets presented in this study can be found in online repositories. The names of the repository/repositories and accession number(s) can be found below: https://github.com/tilsen/TiR-model.git.

## Author contributions

The author confirms being the sole contributor of this work and has approved it for publication.
