# Supplementary material for: An informal logic of feedback-based temporal control
Source: Front Hum Neurosci. 2022 Jul 29;16:851991. doi: 10.3389/fnhum.2022.851991 (PMC9372483; doi:10.3389/fnhum.2022.851991)
Supplement: Supplementary file 1 [file Data_Sheet_1.PDF]

Supplementary Material - Model details

These supplementary materials provide detailed information regarding the mathematical and computational implementation of the model used for all simulations in the paper, "An informal logical of feedback-based temporal control". The conceptual motivation for various aspects of the model is discussed as well. The model described below (with corresponding MATLAB code available at https://github.com/tilsen/TiR-model), was designed to be as simple as possible while still having the ability to generate a variety of empirical phenomena. I expect that there are ways I am unaware of in which the model could be simplified, as well as important phenomena which the model cannot generate without revision.

Model analogies

The development of model was guided by physical analogies. The purpose of describing these analogies is not to justify them, nor to argue that they are better than other analogies. Rather, describing the analogies may be useful to readers who wish to gain further insight into the mathematical details of the model. This should facilitate revision, extension, and criticism of the model. I do not claim that these analogies are empirically well motivated or consistent with each other, but I do assume that they have some degree of internal consistency and utility.

It is important to recognize that in mathematical models of cognitive systems, the systems that we model are always hypothetical constructs. We cannot observe these systems in any direct way (in the sense that we can "directly observe" a ball rolling down a hill, changes in the transmembrane potential of a neuron, or the aperture between the lips). Since we cannot observe our hypothetical constructs directly, we must develop a conceptual model of them based on analogies. More specifically, analogies are sets of conceptual metaphors (Lakoff, 1993), and conceptual metaphors are mappings from a relatively abstract domain (such as cognition) to a relatively concrete domain (such as physical phenomena). Thus our all of models of cognition are based on sets of conceptual metaphors.

The mechanical body analogy

One important example of a physical analogy occurs in the Task Dynamic (TD) model of speech production of Saltzman & Munhall (1989), which is an outgrowth of earlier models of motor behavior in other domains (e.g. Turvey 1977 and Kelso & Holt 1980). The analogy relates specifically to the interactions between gestural systems and vocal tract (VT) systems. Here are the key metaphoric mappings of the analogy, where entities/relations in the abstract domain are *italicized* and entities in the physical domain are **bolded**:

ABSTRACT DOMAIN (cognition)		CONCRETE DOMAIN (physical phenomena)
• *vocal tract systems* (a.k.a tract variables)	ARE	**mechanical bodies (objects)**
• vocal tract system *states*	ARE	**positions and velocities**
• gesture-to-vocal tract system *interactions*	ARE	**mechanical forces**

It is worth pointing out that vocal tract systems are *not literally* mechanical bodies. Rather, they are hypothesized cognitive systems that control geometric dimensions of the vocal tract, such as lip aperture (LA), which is also not a mechanical body. There is no sensible way in which VT systems can be construed as *actually* being mechanical bodies, and so the mappings above are undoubtedly metaphors.

The state of a VT system is a position/velocity vector $[x, \dot{x}]$. The TD model equation for a VT system can also describe a mechanical body, namely a damped, driven harmonic oscillator. The equation can be written as:

$$\ddot{x} = -\beta_G\dot{x} - k_G x + k_G T_G$$

On the left hand side of the equation is an acceleration, with implicit mass $\mathrm{m} = 1$. On the right hand side are three "forces". The term $\beta_G\dot{x}$ is a damping force (analogous to friction) that counteracts rapid accelerations/decelerations. The term $k_G x$ is a restoring force, which restores the system to an equilibrium position of 0. The term $k_G T_G$ is a driving force. Thus the equation can be seen to be an instance of the Newtonian law: $F = \mathrm{m}a$, or to match the form of the equation above:

$$\mathrm{m}a = F_{\text{damping}} + F_{\text{restoring}} + F_{\text{driving}}$$

Note that stipulating an "implicit" mass of $\mathrm{m} = 1$is an heuristic strategy that is adopted because we do not know how to differentiate our systems with respect to this parameter. If we wish to maintain the physical analogy to a damped driven harmonic oscillator, it does not make sense to say that our systems are "massless"--rather, our systems have an invariant mass and we do not differentiate their masses. Indeed, the mass parameter is always implicitly there, as it scales the values all of the other parameters of the equation.

The subscripts G on the parameters of the damping, restoring, and driving forces are included to represent the fact that all three parameters (and hence all three forces) are functions of time-varying gestural activation:

- $\beta_G = f_\beta(G(t))$
- $k_G = f_k(G(t))$
- $T_G = f_T(G(t))$

Indeed, much of the complexity in the Saltzman & Munhall (1989) model arises from developing rules to determine how these parameters vary as a function of the activation states of gestural systems.

Although the analogy between VT systems and mechanical systems is clear, it does not apply to gestural systems. Gestural systems in the Saltzman & Munhall (1989) model are implemented as hand-specified step functions in a unit-normalized activation coordinate. Thus the state space of gestural systems is a one-dimensional variable in the range $[0, 1]$, which is referred to as "activation". This notion of activation does not gel with the mechanical system analogy. With regard to the construction of the three gesture-dependent forces--$\beta_G\dot{x}$ (damping), $k_G x$ (restoring), and $k_G T_G$ (driving)--the activation states of gestures play complicated roles in determining the "strengths" of the forces. Gestural activation states are not equivalent to or even necessarily proportional to the strengths of any of one these forces. This is due to the fact that, when multiple gestures are active, their contributions to each force are blended in a way that makes the "force strength" interpretation hard to maintain. Furthermore, we cannot interpret gestural systems as mechanical ones (like VT systems), because the states of gestural systems are not velocity and position ($[\dot{x}, x]$) vectors. This raises the question: is there a useful physical analogue for the activation states of gestural systems?

The thermodynamic system analogy

I think that a useful analogy exists, and that it applies both to gestural systems and to most of the TiR systems that I have described in the manuscript:

ABSTRACT DOMAIN (cognition)		CONCRETE DOMAIN (physical phenomena)
• gesture and TiR systems	ARE	**thermodynamic systems**
• gesture and TiR system *states* (activations)	ARE	**thermodynamic energies**
• gesture-TiR *interactions*	ARE	**energy transfers**

Why are thermodynamic systems useful analogies for gestures and TiRs? My reasoning is based on the admittedly speculative idea that each of these systems is physically instantiated by a relatively large population of neurons which may be located in one or more areas of the central nervous system. The exact locations of these populations and their degrees of overlap (independence) are potentially important but not directly relevant to the gist of the analogy.

Given the idea that each system corresponds to a large population of neurons, it is relevant to first consider how we might conceptualize even a single neuron within a population. I note that my understanding of the physiology and dynamics of neurons is heavily influenced by the work of Izhikevich (2007). Also, there is a vast amount of detail which is omitted from the following.

We can think of an individual neuron as a system which is separated from its surroundings, with the separation being accomplished by a cell membrane. The membrane has various types of ion gates/pumps, and these serve to maintain electrical and chemical gradients. Both of these types of gradients are associated with forces that influence the flux of ions through the membrane. The "resting state" of the neuron corresponds to a balance between these forces. Some types of neurons have the property that the resting state is close to a critical state (threshold) at which the neuron undergoes a phase transition, i.e. a rapid depolarization (spike). This can be viewed as an instance of self-organized criticality in the sense of Bak (1988): the neurons are systems that, when embedded in the appropriate surroundings, evolve toward a critical state. In other words, the equilibria of some types of neurons are states in which they are "ready" to depolarize.

One cause of the phase transition (rapid depolarization) can be changes in the transmembrane gradients that are caused by depolarization of other neurons, via synaptic mechanisms. When a pre-synaptic neuron depolarizes, it releases neurotransmitter molecules at its synaptic junctions, and this causes changes in the ion gates of the membrane of a post-synaptic neuron. Hence it is reasonable to say--metonymically--that one neuron "exerts a force" on another, since the pre-synaptic neuron transitions to state in which it causes a change in the gradients associated with the post-synaptic neuron. A more neutral statement might be that a change in the state of a pre-synaptic neuron is associated with a change in the forces that a post-synaptic one experiences from its surroundings.

Furthermore, an important property of some neurons (including the "regular-spiking" cortical layer 5 pyramidal neurons described by Izhikevich (2007: 372)), is that in the resting state they respond monotonically to weak input (forces which are not sufficiently strong to cause a depolarization in and of themselves). This means that the more input these neurons receive, the closer they get to the threshold for spiking. A highly idealized "leaky integrate-and-fire" dynamics is described in Izhikevich (2007: 358), which has some of the important properties of neuronal spiking. This very simple model is:

$$\dot{v} = b - v, \quad \text{if } v = 1, \text{ then } v \leftarrow 0$$

where v represents the membrane voltage, and b is the input current, which can vary over time. The term $-v$ is the leak current. A spike occurs when $v = 1$.

This is not an adequate model of any neuron, since it does not capture the dynamics of the spike or repolarization, but it does exhibit the important properties of (i) having a voltage state, (ii) having a threshold, and (iii) generating some event when the threshold is reached. It is not difficult to extend this very simple model to capture the dynamics of the spike, but for current purposes the idealized model will suffice.

Macroscopic construction of state variables

Now let's imagine that the large population of neurons that we assume to be associated with a gestural system is a collection of these leaky-integrate-and-fire neurons. The neurons within the population "receive excitatory input from" (experience depolarizing forces caused by) neurons associated with other populations--these are "external" forces because they are from neurons outside of the population. The neurons within the population also experience "internal" forces from each other. Given this situation, how should we conceptualize and model the state of the population? A very simple approach is to imagine that we can count the number of depolarization events that occur over a short period of time, summing over the entire population--a *spike rate*. Because we are dealing with a very large population, we can think of the spike rate as an approximately continuous scalar variable. This variable is bounded below at 0 (no spiking), and is bounded above in a way that depends on population size and neurophysiological constraints on how frequently a neuron can generate a spike.

Hence the system state--which we call "activation"--is a spike rate, a short-term integration of spikes, and it has units of $\frac{\text{spikes}}{\text{time}}$.

But what do "spikes" correspond to, in a physical units? In the integrate-and-fire model, they are events that begin when voltage reaches a threshold value of 1, and they end with the return of that voltage to a resting value of 0. This means that spikes are stereotyped changes in voltage over a short time period, with units of $\left(\frac{\Delta V}{\Delta t}\right)$. Hence our system state variable of spike rate (activation) can be conceptualized as a time-integral of changes of voltage (V) over time, i.e.

activation (system state) = $\int \left(\frac{\Delta V}{\Delta t}\right) dt$

Furthermore, consider that voltage is the ratio of work to charge: $V = \frac{W}{Q}$, and has dimensions:

$ML^2T^{-2}Q^{-1}$

where:

- M is mass
- L is length or distance
- T is time
- Q is charge

Therefore, activation also has the dimensions of a voltage, i.e. an amount of work per charge:

$\int \left(\frac{\Delta V}{\Delta t}\right) dt$ has units of: $ML^2T^{-2}Q^{-1}$

Now we are faced with a similar situation as occurred in the mechanical analogy for vocal tract systems: we do not know how to distinguish our gestural systems with respect to charge, just as we did not know how to distinguish are vocal tract systems with respect to mass. So, we will adopt a heuristic stipulation that $Q = 1$, just as we posited that $m = 1$ for the damped driven harmonic oscillators. Thereby, through nondimensionalization of charge, our activation state for gestural systems and TiRs can be expressed in dimensions of energy:

$E = ML^2T^{-2}$

In other words, in the thermodynamic analogy, gestural system and TiR system states are energies. Moreover, given that these energies represent the collective behaviors (spikes) of many individual component systems (neurons), it is appropriate to adopt a thermodynamic/statistical interpretation of our system. A change in the internal energy of a thermodynamic system is typically conceptualized as the sum of products of conjugate pairs of generalized forces and generalized fluxes (or displacements), i.e.

$\Delta E = TdS - PdV + \mu dN$ (+ more force/flux conjugates)

Each conjugate pair represents a different way in which energy can be transferred to or from the system. Specifically, the term PdV is mechanical work, with pressure P considered a generalized force and change in volume dV a generalized displacement. The term TdS is heat energy, with temperature being the generalized force and change in entropy the generalized displacement. The term μdN is material energy transfer (i.e. energy transferred through exchange of matter), with the chemical potential μ of particles/molecules being the force and the number N of particles/molecules being the flux. There are additional conjugates we might add to the above equation, including stress-strain work, electrical work, surface work, gravitation work, electric polarization, and magnetic polarization.

It is an open question whether some of these various types of physical energy transfer are more useful than others for conceptualizing the interactions between neural populations. For the purposes of constructing a simple model, there is no need to commit to one particular interpretation of the energy transfer (and yet, doing so may be useful for developing more powerful models). Nonetheless, there is a crucial point to make about how we should conceptualize the energy transfer in the case of interacting neural populations. This point is important enough to be considered a "principle" of the analogy:

The principle of indirect energy transfer between neural populations:

- An action of system A on system B causes energy to be transferred between B and its surroundings , $\mathcal{S}_B$.

The indirect transfer principle tells us that energy is not transferred directly between systems. Such would be inconsistent with our microscopic understanding of neuronal signaling, which does not involve the direct transfer of ions from one neuron to another. Instead, when two populations of neurons interact, we think of the interaction as indirect energy transfers: the interactions cause energy to be transferred between the neurons in a population and their surroundings. Because we do not model the surroundings, we cannot readily apply the first law of thermodynamics (energy conservation) to reason about our systems and their interactions. The

systems are partly "open" to their surroundings. Furthermore, the "forces" that we incorporate in our model of system interactions are metonymic: the forces that B experiences come directly from its surroundings, $\mathcal{S}_B$, while system A is what causes the surroundings $\mathcal{S}_B$ to exert force on B.

Macroscopic extension of integrate-and-fire dynamics

Now that we have a conceptual model of our system state variable (x is an energy), we need to construct a dynamics for that variable:

$$\frac{dx}{dt} = ?$$

Our construct is based on applying the very simple idealization of the leaky integrate-and-fire model of a single neuron, described above. In a sense, we are asserting that the dynamical behavior observed for each of the many microscopic components of our system (which we do not model) emerges macroscopically in the collective behavior of those components. On the macroscale, this behavior describes changes in the "activation" (energy) of the population. Recall that the microscale equation was:

$$\dot{v} = b - v, \quad \text{if } v = 1, \text{ then } v \leftarrow 0$$

For the macroscale, we will adopt an even simpler idealization in which we ignore the leak term ($-v$). (To justify this, we might speculate that chains of interactions between neurons within a population partly counteract the leak currents of individual neurons.) At the same time, we will parameterize the threshold to τ, and rather than resetting the system to 0 when it reaches the threshold, we will construct a event $\mathscr{A}$, which can be read as "act on other systems" and which entails that the system can exert a force on other systems. Renaming v to x, and renaming b to $F(t)$, we have:

$$\frac{dx}{dt} = F(t), \quad \text{if } x = \tau, \text{ then } \mathscr{A}$$

The time-dependent function $F(t)$ is the total force experienced by a system per unit of time, and it represents the sum of the generalized forces associated with various energy transfers to the system. These component forces are usually associated with the actions $\mathscr{A}$ of other systems in the model. Expressing the equation in a way that shows how we obtain a numerical solution clarifies that the energy Δx transferred in each time step of the model simulation is a product of the force per unit time $F(t)$ and the time step Δt:

$$\Delta x = F(t)\Delta t$$

Note that we have adopted the heuristic of treating the conjugate generalized displacements of the forces as having values of 1. Hence we can say that the system state variable (x, an activation) is an "integration of force" that the system experiences over time. It is more precisely correct to say that the system integrates the energy transferred over time. One reason that we refer to integration of force rather than energy is that it is only the force that varies, while the flux/displacement is nondimensionalized. In the general equation for system dynamics presented below, changes in system states are a sum of the energy transfers, each of which can be conceptualized as products of forces with implicit fluxes/displacements.

Model equations

Different types of systems in the model experience different sets of forces. Indeed, system types can be distinguished according to the sources of force that they experience. Nonetheless, it is useful for simplifying the computational implementation of the model to construct a general equation for all system dynamics. In this equation, the change in the state of any system is equated to a sum of forces. However, in most cases only one or two of these forces is ever experienced by a given system, with the remaining ones being zero. The general equation of the model is:

Eq. (1): $$\frac{dX}{dt} = F_{\mathcal{S}}(X,\Omega) + F_r(X) + F_\Phi(X,\Phi) + F_\alpha(X,\alpha) + F_\sigma(X) + F_\chi(X,\chi,\tau)$$

where X is a vector of system state variables, and F are various forces, listed below:

$F_{\mathcal{S}}(X,\Omega)$: gated autonomous growth

- Ω: non-autonomous growth rates / frequencies

$F_r(X,k)$: oscillator amplitude dynamics

- k: stiffnesses of oscillators

$F_\Phi(X,\Phi)$: oscillator phase dynamics

- Φ: phase coupling force magnitudes and signs

$F_\alpha(X,\alpha)$: non-autonomous TiR integration / gestural gating forces

- α: integration rates

$F_\sigma(X,\sigma)$: non-autonomous suppression / degating forces

- σ: suppression force strengths

$F_\chi(X,\chi,\tau)$: action forces

- χ: action valence (gating/degating)
- τ : action threshold (specified in units of x)

For any particular system, most of the forces included in Eq. (1) are always zero, and so Eq. (1) can be reduced in various system-specific ways, described below.

Types of systems

The following is a list of different types of systems which can be included in the model. System types as named in the model Matlab code are shown in parentheses, along with symbols commonly used in schemas. Also shown are state space limits or modulus values when applicable:

Gestural systems, gestural gating systems, and sensory systems:

- Gestural systems (*gest,* g_1): $[0, 1]$
- Gestural gating systems (*gate,* G_1): $[0, 1]$
- Sensory systems (*sens,* g'_1): $[0, 1]$

Non-autonomous TiRs:

- Intra-gestural TiRs (*itmr,* $\widetilde{T}_1$ or $\widetilde{1}$ or $\sim$): $[0, \infty)$
- Inter-gestural TiRs (*etmr,* $\widehat{T}_1$ or $\hat{1}$ or $\wedge$): $[0, \infty)$
- Extra-gestural TiRs (s*tmr,* $\overline{T}_1$ or $\overline{1}$ or $-$): $[0, \infty)$

Autonomous TiRs:

- Periodic TiR phases (*osc,* θ_1): modulus 2π
- Periodic TiR radial amplitude (*oscr1*): $[0, \infty)$
- Periodic TiR radial amplitude first derivative (*oscr2*): $(-\infty, \infty)$
- Periodic TiR gating systems (*oscg*): $[0, 1]$
- Aperiodic TiR systems (*extr,* ϵ_1): $[0, \infty)$
- Aperiodic TiR gating systems (*exg*): $[0, 1]$

Competitive queuing systems:

- Competitive queuing systems (*cq,* μ_1, ν_1): $[0, 1]$
- Competitive queuing gating systems (*cqg*): $[0, 1]$

Gestural systems

```
M = fbmod_models('external vs internal');
M = run(M);
draw_schema(M);
```

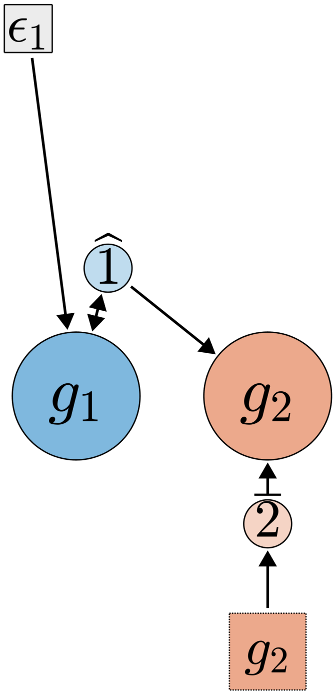

For gestural systems, Eq. (1) can be reduced to:

$$\frac{\delta x_{\text{gest}}(t)}{\delta t} = F_\alpha + F_\sigma$$

Eq. (2): $\Delta x_{i,t} = \Delta t\left(\sum_j \alpha_{ji} x_{j,t-1} + \sum_j \sigma_{ji}[1 - x_{j,t-1}]\right)$

where t is an integer time index. The first sum corresponds to F_α, the gestural degating force, and the second sum corresponds to F_σ, the gestural gating force. α_{ji} is the gate-opening force which the gesture i experiences from gestural gating system j, and σ_{ji} is the gate-closing force. The values of α and σ which correspond to forces exerted by gates are set to $\pm\frac{1}{\Delta t}$ in order to impose the condition that gestural systems evolve to maximum/minimum activation in one timestep. For all simulations reported in the manuscript, each gesture only experiences forces from its unique associated gating system.

```
param_matrix(M,'alpha');
```

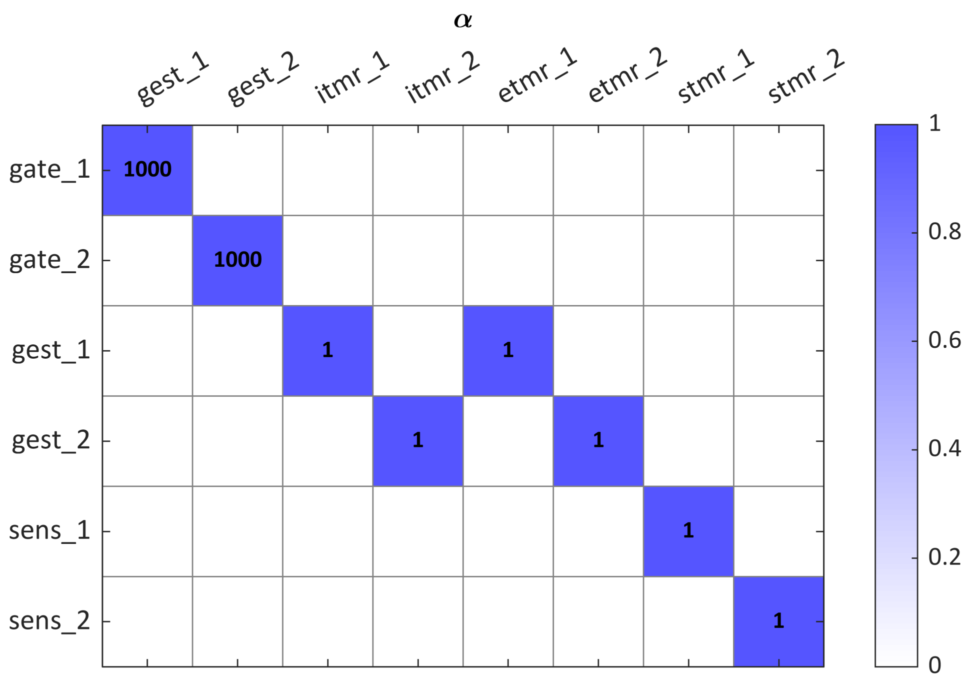


```
param_matrix(M,'sigma');
```

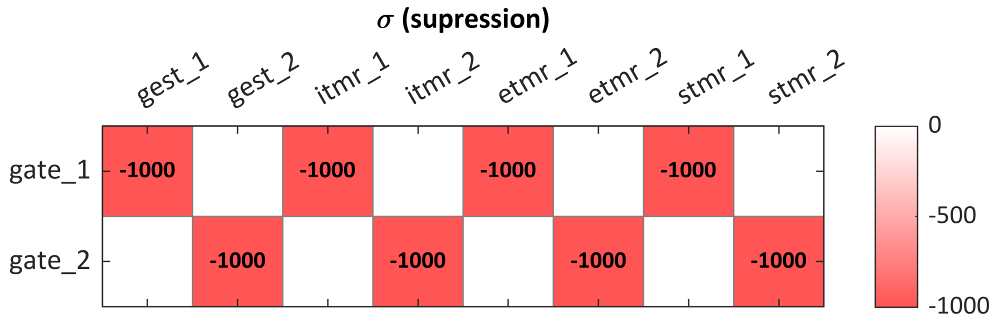

The min and max functions are applied to gestural systems at each time step to constrain their activation values to the interval $[0, 1]$:

$$x_{i,t} = \max\left(\min\left(x_{i,t} + \Delta x_{i,t}, 1\right), 0\right)$$

Gestural gating systems

For gestural gating systems, Eq. (1) reduces to:

Eq. (3): $\Delta x_{i,t} = \Delta t \sum_j F_\chi(x_{j,t-1}, \chi_{ji}, \tau_{ji})$

where $F_\chi(x_j, \chi_{ji}, \tau_{ji})$ is the action of system j on gestural gate i. This action is defined as:

Eq. (4): $F_\chi(x_{j,t-1}, \chi_{ji}, \tau_{ji}) = \chi_{ji} [\![\sqrt{(x_{j,t-1} - \tau_{ji})^2} < \delta_j]\!]$

where the inequality inside $[\![ ]\!]$ evaluates to 1 when true and 0 when false. The parameter δ_j is in effect a tolerance for how close to the threshold a system state must be in order to act on another system. For all aperiodic systems this value was set to $\delta = \dfrac{2}{\Delta t}$ and for all periodic systems this value is set to $\delta = \dfrac{2}{2\pi \max\limits_{j \in osc} \Omega_j}$, where Ω_j are the oscillator frequencies. These settings ensure that systems exert actions when they reach the threshold.

The matrix τ specifies the TiR state values (thresholds) at which TiRs act upon other systems, including gestural gating systems:

```
param_matrix(M,'tau');
```

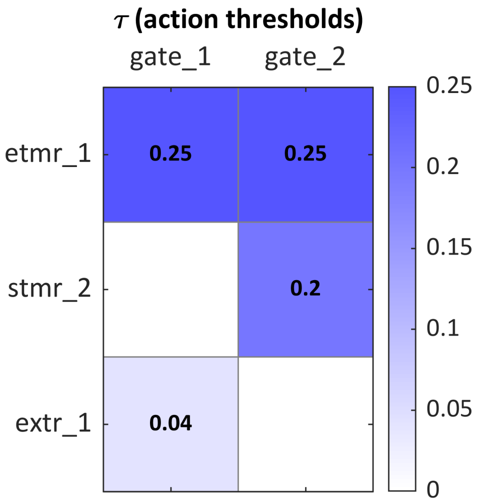

The matrix χ specifies the valence of TiR actions on other systems:

```
param_matrix(M,'chi');
```

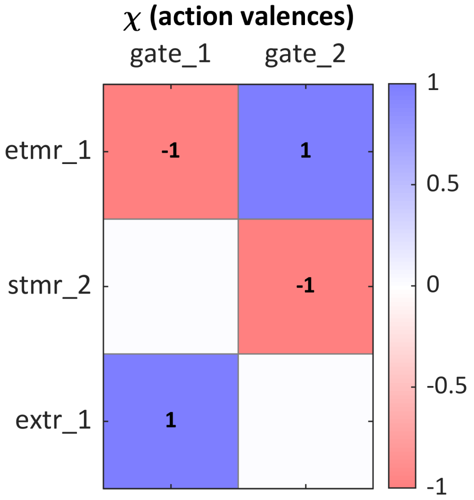

In this example, the thresholds and valences of the actions on gate_1 and gate_2 have the following effects:

- extr_1 opens gate_1 when extr_1 = 0.040
- etmr_1 closes gate_1 when etmr_1 = 0.250
- etmr_1 opens gate_2 when etmr_1 = 0.250
- stmr_2 closes gate_2 when stmr_2 = 0.200

Sensory systems

Sensory system states are modeled as veridical, delayed gestural system states. This is implemented by setting the state of each sensory system to the state of the corresponding gestural system at a fixed time in the past:

Eq. (5): $x_i(t) = x_i'(t - \delta_{\text{sens}})$

where x_i' is the gestural system which corresponds to sensory system x_i, and δ_{sens} is the delay between gestural states and cortical sensory system representations of those states, here assumed to be 0.040 s.

Non-autonomous timekeepers

The matrix α also specifies the forces that gestural systems and sensory systems exert on various non-autonomous TiRs. For non-autonomous TiRs, the system equation is:

$$\frac{\delta x_{\text{TiR}}(t)}{\delta t} = F_\alpha + F_\sigma$$

Eq. (6): $\Delta x_{i,t} = \Delta t\left(\sum_j \alpha_{ji} x_{j,t-1} + \sum_j \sigma_{ji}[1 - x_{j,t-1}]\right)$

which is identical to the gestural system equation (Eq. (2)). However, examining the matrix α shows that intra-gestural (itmr), inter-gestural (*etmr*), and extra-gestural (*stmr*) TiRs differ from gestural systems in that they integrate forces experienced from gestures or sensory systems. Specifically, intra-gestural and inter-gestural TiRs (*itmr*, *etmr*) integrate forces from gestures, and external TiRs (*stmr*) integrate forces from sensory systems.

Aperiodic autonomous timekeepers and gating systems

Autonomous TiRs (*extr*), unlike non-autonomous TiRs, have gating systems that can control when their activation states begin to increase and can halt the increase of their activation.

```
M = fbmod_models('auto vs nonauto');
M = run(M);
draw_schema(M);
```

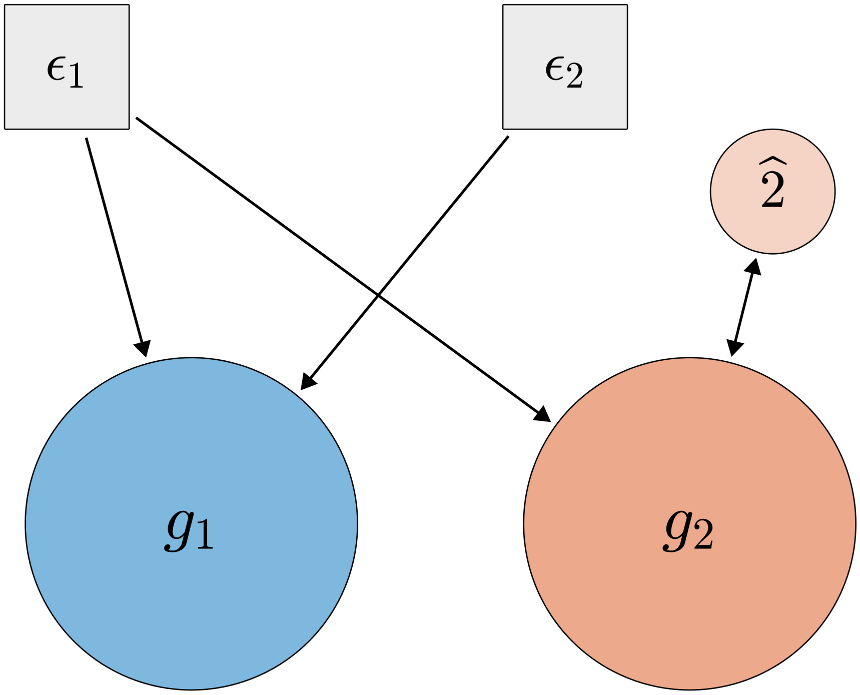


```
param_matrix(M,'omega');
```

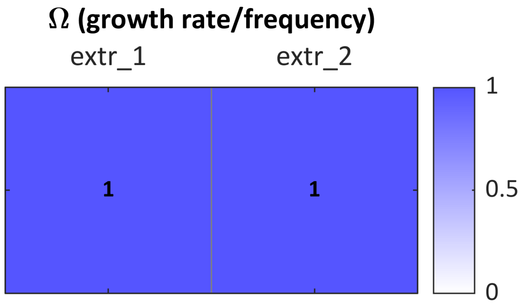


```
param_matrix(M,'map_exg_extr')
```

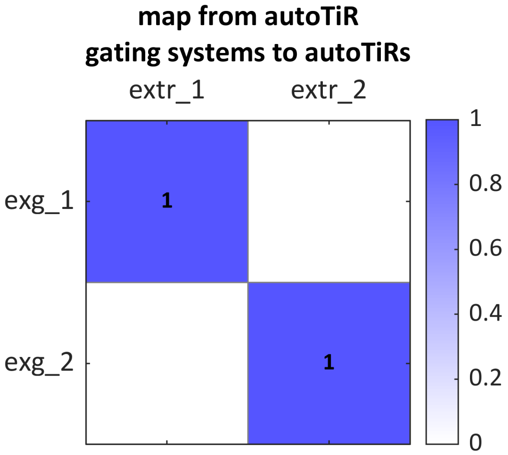

Aperiodic autonomous TiRs are described by the following equation:

$$\frac{\delta x_{\text{autoTiR}}(t)}{\delta t} = F_{\Omega}$$

Eq. (7): $\Delta x_{i,t} = \Delta t \cdot \omega_i \cdot x^G_{i,t-1}$

where ω_i is the intrinsic growth rate, and x^G_i is the (binary) state of the associated gating system.

Periodic autonomous timekeepers (coupled oscillators)

For the phase variables of coupled oscillators, Eq. (1) reduces to :

$$\frac{\delta x_{\text{osc}}(t)}{\delta t} = F_{\omega} + F_{\Phi}$$

Eq. (8): $\Delta x_{i,t} = \Delta t \left(\omega_i \cdot x^G_{i,t-1} + \sum_j \phi_{ji} \sin(x_{i,t-1} - x_{j,t-1})\right)$

where ω_i is the intrinsic oscillator frequency in units of radians/s, and x^G_i is the (binary) state of the associated gating system. The sum is a sum over relative phase coupling forces.

For the amplitude (x_r) and amplitude derivative ($x_{\dot{r}}$) variables of coupled oscillators, Eq. (1) reduces to:

$$\frac{\delta x_r(t)}{\delta t} = F_r = x_{\dot{r}}$$

$$\frac{\delta x_{\dot{r}}(t)}{\delta t} = F_r = -2\sqrt{k}\, x_{\dot{r}} - k(x_r(1-\beta))$$

Eq. (9): r: $\Delta x_{i,t} = \Delta t \cdot x^{\dot{r}}_{i,t-1}$

$\dot{r}$: $\Delta x_{i,t} = \Delta t \cdot \left(-2\sqrt{k}\, x_{i,t-1} - k(x^r_{i,t-1} \cdot [1-\beta])\right)$

where $x_i^{\dot{r}}$ is the radial amplitude derivative corresponding to radial amplitude x_i and conversely x_i^{r} is the radial amplitude corresponding to radial amplitude derivative x_i. The term $-2\sqrt{k}$ corresponds to critical damping. The equilibrium amplitude $\beta = 1$ and stiffness $k = 5000$ for all oscillators.

```
M = fbmod_models('oscillators_ggg');
M = run(M);
draw_schema(M);
```

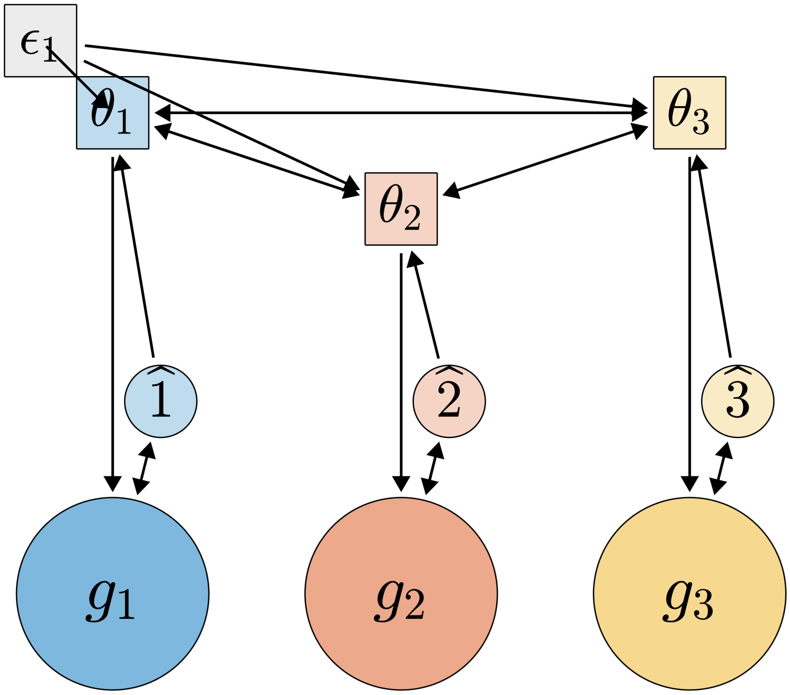


```
M.freq = M.omega/(2*pi); %convert to Hz

param_matrix(M,'freq');
```

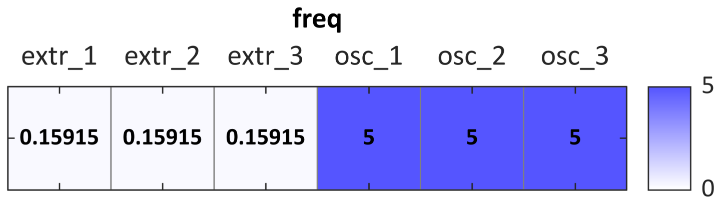


```
param_matrix(M,'tau');
```

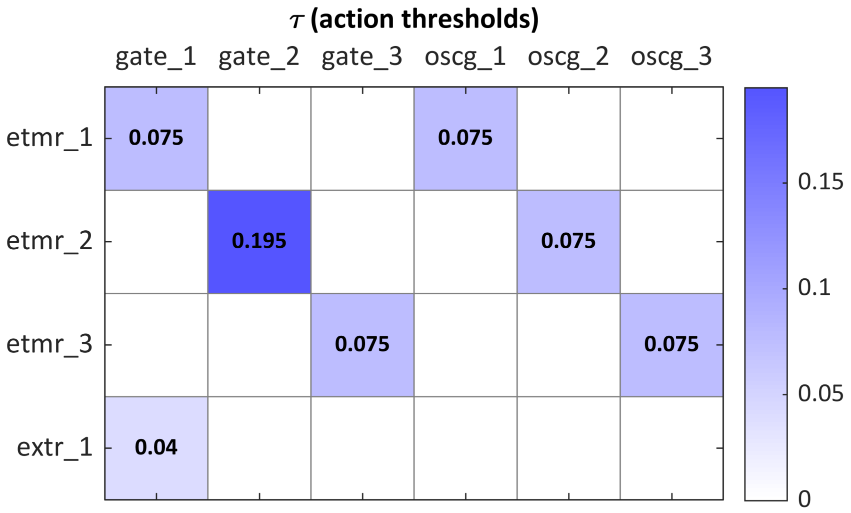

The actions that oscillator systems exert on gestural gates depend both on oscillator phase, as specified in threshold matrix τ, and on oscillator amplitude. The condition is imposed that oscillators only act on gestural system when their amplitudes exceed a threshold value of 0.5. This condition is implemented in the Matlab code by setting the input of an oscillator to the action force function to a non-numeric value when its radial amplitude is below 0.5.

Competitive queuing systems and gates

Competitive queuing systems (cq), along with their gates (cqg) are a special class of TiRs which can be grouped into competitively selected sets of systems. The cq systems which comprise a group are selected in a mutually exclusive way--only one system can be selected at a time. The implementation of mutual exclusion is accomplished with a matrix which maps from a cq system to the gating systems of all of the cq systems with which it interacts:

```
X = load(['fbmod_multilevel_competitive_selection.mat']);
M = X.M; %model
U = X.U; %mu systems in the utterance
P = X.P; %conceptual (prosodic word) systems

param_matrix(M,'map_cq_cqg')
```

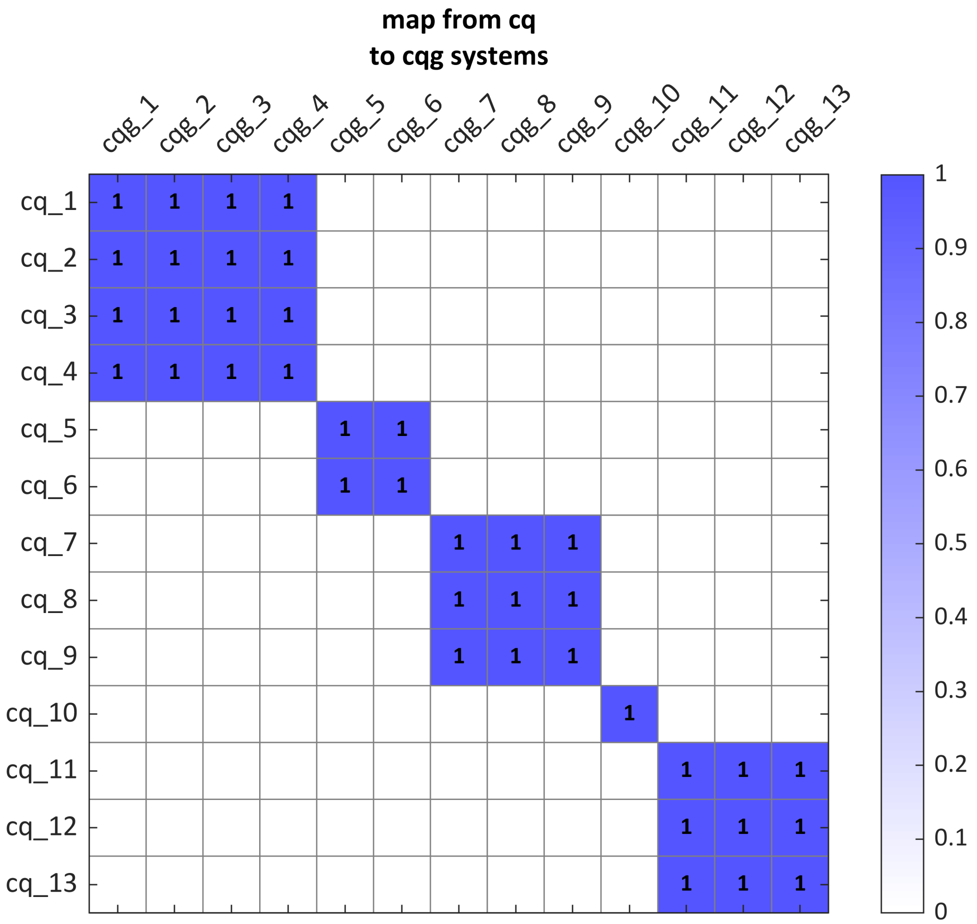

Competitive queuing gate states are controlled with the following discrete logic. In each timestep, for each cq/cqg system, the following logical conditions are calculated:

- condition c^1: any cq system in the associated set is selected (has a value ≥ 1)
- condition c^2: all cq systems in the associated set have previously been selected.
- condition c^3: the higher level cq system (if present) which governs this cq system is not selected

The states of all cqg systems (gates) are then operated on, such that the gate is closed if any of the three conditions holds, otherwise it is opened:

$$x_{\text{cqg}} = \begin{cases} 0, & c^1 \vee c^2 \vee c^3 \\ 1, & \neg(c^1 \vee c^2 \vee c^3) \end{cases}$$

The relations between higher level cq systems (C-systems) and lower level ones (μ-systems) are specified in map from higher-level cq systems to lower level ones:

```
param_matrix(M,'map_cq1_cqg0')
```

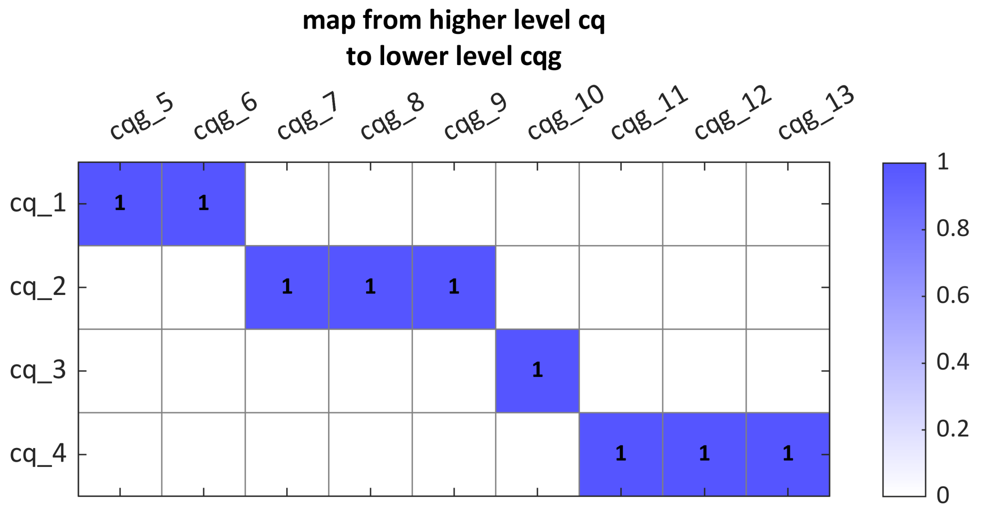

An outstanding challenge in further development of the model is to convert the discrete/logical handling of competitive selection dynamics to a fully continuous/dynamical one.

Simulation details

All simulations reported in the manuscript were conducted with a timestep of $\Delta t = 0.001$s, i.e., 1 ms. Simulation durations were adjusted to allow sufficient time for all gestures to be activated and deactivated. TiR action-threshold parameters were set to generate empirically reasonable gestural activation dynamics. Oscillator frequencies were 5.0 Hz unless otherwise specified.

Effects of global and local noise for three-gesture systems

For the analysis of covariance between initiation intervals in three-gesture systems, 400 simulations were conducted for each of 25 combinations of local and global noise levels. The noise was implemented as follows.

For oscillator frequency (ω) noise, in each simulation random numbers were drawn from uniform distributions in the range $[-\nu, +\nu]$and were added to the base frequency 5 Hz. Values of ν were varied in five steps from 0 (min noise amplitude) to 2.5 Hz (max noise amplitude). Hence the actual frequencies of each oscillator were:

$$\omega_i = \omega^{\text{base}} + \omega^{\text{global}} + \omega_i^{\text{local}}$$

For non-autonomous TiR integration rate (α) noise, in each simulation random numbers were drawn from Gaussian distributions ($\mu = 0,\ \sigma_\alpha$) and were added to the base α of 1.0. Values of σ_α were varied in five steps from 0 (min noise amplitude) to 0.2 (max noise amplitude). Hence the actual integration rates of the non-autonomous TiRs were:

$$\alpha_i = \alpha^{\text{base}} + \alpha^{\text{global}} + \alpha_i^{\text{local}}$$

It should be noted that when global and local oscillator noise levels are high, oscillator frequencies can become substantially different from each other, leading to chaotic phase evolution and disordered gestural triggering. These cases were excluded from analyses of noise effects on covariance.

Effects of self-attention variation

For the simulation of variation in self-attention, represented by λ, the self-attention parameter λ was varied in 21 steps over the range $[0, 1]$, and α parameters for non-autonomous TiRs were modulated as follows:

$$\alpha(\lambda) = \frac{\alpha_{\text{base}}}{1 + \beta\lambda}$$

with $\beta_{\text{stmr}} = 2$ for external/sensory TiRs, $\beta_{\text{etmr}} = 1$ for internal TiRs, and $\alpha_{\text{base}} = 1$.

Effects of pace variation

For the simulation of variation in pace, represented by λ, the pace parameter λ was varied in 21 steps over the range $[0, 1]$, and ω parameters for coupled oscillators were modulated as follows:

$$\omega(\lambda) = \omega_{\text{base}} - \omega_{\text{range}} \tanh\left(\lambda^{\text{n}}\left(\lambda - \frac{1}{2}\right)\right)$$

where $\omega_{\text{base}} = 6$ Hz, $\omega_{\text{range}} = 8$ Hz, $\tanh$ is the hyperbolic tangent function, and $\lambda^{\text{n}} = 5$ controls the nonlinearity in the mapping between λ and ω.

Effects of selectional anticipation

The effect of selectional anticipation associated with selection of the final set of systems out of set of competitively selected systems was simulated by imposing:

$\beta_{\text{stmr}} = 2.0$, $\beta_{\text{etmr}} = 1.0$ for non-final sets

$\beta_{\text{stmr}} = 2.2$, $\beta_{\text{etmr}} = 1.2$ for the final set

The effects of self-attention, pace variation, and selectional anticipation described above were applied in the simulations of speech rate variation for 11 steps of λ over the range $[0, 1]$.

References

- Bak, P., Tang, C., & Wiesenfeld, K. (1988). Self-organized criticality. *Physical Review A*, *38*(1), 364.
- Izhikevich, E. M. (2007). *Dynamical systems in neuroscience*. MIT press.
- Kelso, J. A. S., & Holt, K. G. (1980). Exploring a vibratory systems analysis of human movement production. *J Neurophysiol*, *43*(5), 1183–1196.
- Lakoff, G. (1993). *The contemporary theory of metaphor*.
- Saltzman, E., & Munhall, K. (1989). A dynamical approach to gestural patterning in speech production. *Ecological Psychology*, *1*(4), 333–382.

- Turvey, M. T. (1977). Preliminaries to a theory of action with reference to vision. *Perceiving, Acting and Knowing*, 211–265.


```
function [M] = run(M)
M = fbmod_run_models(M,'verbose',false,'parallel',false);
M = fbmod_prep_schema(M);
end

%%
function [] = draw_schema(M,varargin)
h = fbmod_helpers;
figure;
if nargin==1
    fbmod_draw_schema(M,h,...
        'linewidth',1,'reduce_lines',[0.005 0.005]);
elseif strcmp(varargin{1},'large')
    fbmod_draw_schema(M,h,...
        'linewidth',1,'reduce_lines',[0.005 0.005]);
end

end

%% plots parameter matrix:
function [] = param_matrix(M,param)
plot_transform = {};
titlestr = param;
fontsize = 11;
switch(param)
    case 'alpha'
        plot_transform = @(x)x~=0;
        titlestr = '\alpha';
    case 'tau'
        titlestr = '\tau (action thresholds)';
    case 'chi'
        titlestr = '\chi (action valences)';
    case 'sigma'
        titlestr = '\sigma (supression)';
    case 'omega'
        titlestr = {'\Omega (growth rate/frequency)'};
    case 'map_exg_extr'
        titlestr = {'map from autoTiR','gating systems to autoTiRs'};
    case 'map_cq_cqg'
        titlestr = {'map from cq','to cqg systems'};
    case 'map_cq1_cqg0'
        titlestr = {'map from higher level cq','to lower level cqg'};
end
fbmod_plot_system_matrix(M,param,...
    'fontsize',fontsize,...
    'fontsize_values',9,...
    'show_values',true,...
    'plot_transform',plot_transform);
```

```
title(titlestr,'fontsize',fontsize);
axis equal;
axis tight;

fh = gcf;
axh = gca;

resize_figure(fh,axh);

end

%% resizes parameter matrix figure for nicer export
function [] = resize_figure(fh,axh)

%add some extra space at top and bottom by compressing axes
axh.Position([1 3]) = axh.Position([1 3]) + [0.05 -0.05];
axh.Position(4) = axh.Position(4) - 0.05;

nc = diff(get(axh,'XLim'));
nr = diff(get(axh,'YLim'));

%put matrix near bottom of figure
axh.Position(2) = 0.02;

%resize according to rows and columns
fh.Position(3) = min(40*(nc+6),600);
fh.Position(4) = min(40*(nr+4),600);

end
```
